# Community effect of cardiomyocytes in beating rhythms is determined by stable cells

**DOI:** 10.1038/s41598-017-15727-5

**Published:** 2017-11-13

**Authors:** Tatsuya Hayashi, Tetsuji Tokihiro, Hiroki Kurihara, Kenji Yasuda

**Affiliations:** 1 Graduate School of Mathematical Sciences, the University of Tokyo, 3-8-1 Komaba, Tokyo, 153-8941 Japan; 2 CREST, Japan Science and Technology Agency, 4-1-8 Honcho, Kawaguchi, Saitama 332-0012 Japan; 3 Graduate School of Medicine and Faculty of Medicine, the University of Tokyo, 7-3-1 Hongo, Tokyo, 113-0033 Japan; 4 Faculty of Science and Engineering, Waseda University, 3-4-1 Okubo, Tokyo, 169-8555 Japan

## Abstract

The community effect of cardiomyocytes was investigated *in silico* by the change in number and features of cells, as well as configurations of networks. The theoretical model was based on experimental data and accurately reproduced recently published experimental results regarding coupled cultured cardiomyocytes. We showed that the synchronised beating of two coupled cells was tuned not to the cell with a faster beating rate, but to the cell with a more stable rhythm. In a network of cardiomyocytes, a cell with low fluctuation, but not a hight frequency, became a pacemaker and stabilised the beating rhythm. Fluctuation in beating rapidly decreased with an increase in the number of cells (*N*), almost irrespective of the configuration of the network, and a cell comes to have natural and stable beating rhythms, even for *N* of approximately 10. The universality of this community effect lies in the fluctuation-dissipation theorem in statistical mechanics.

## Introduction

Synchronisation of biological cycles is indispensable to life^1,2^. The heartbeat is the representative phenomenon of synchronisation in physiology in which spontaneous pulsations of cardiomyocytes are tuned to a certain beating rate. Extensive research has been devoted to understanding the mechanism of regularity in beating of cardiac cells experimentally and theoretically^3–9^. Contraction of a cardiomyocyte is caused by complex electrophysiological processes. Detailed analyses of these processes requires elaborated mathematical models composed of a large number of equations^10,11^. However, to understand synchronisation, a small number of simultaneous ordinary equations of membrane currents and action potentials, such as the Hodgkin-Huxley equation or its reduced form, the FitzHugh-Nagumo equation and the Van der Pol equation, are sufficient for capturing the main phenomenon of cellular dynamics (see, for example^12,13^). Most mathematical models for interacting cardiomyocytes are based on these equations^14–18^. A network of cardiomyocytes is regarded as a system of interacting, self-sustained (nonlinear) oscillators. To explain the details of synchronisation in such a system of oscillators, phase equations have been extensively and successfully used^19,20^. A variety of studies using phase equations in a network of cardiac cells has been reported, such as synchronisation of cardiac pacemaker cells to external periodic stimuli, phase resetting properties of cardiac cells^21^, and oscillation regularity depending on the cell networks^22^. Analysis methods of cellular neural networks have been extensively studied and some important and interesting results have been obtained^23–25^. Hamada *et al*.^26^ investigated the spontaneous order in synchronisation of beating. They modelled a cell by the WJG model. This model is expressed as fairly complex, simultaneous, differential equations in terms of the membrane potential, ion currents and ion concentrations, and its extension with stochastic processes. They showed that the time interval necessary for synchronisation is strongly dependent on the strength of cell-to-cell conductance, and it is shortened by stochastic fluctuations. Fluctuation of interbeat intervals decreases as the size of the cell cluster increases, which is consistent with the experimental data.

Cells acquire a function of a tissue by forming a group. An example of this situation is that the sinoatrial node, which generates the stable beating rhythm of the normal heart, is composed of cardiomyocytes beating autonomously. This means that the function of cardiomyocytes changes from a cellular level to an organizational level by the effect of the assembly. Investigation into the process of acquiring function helps us to understand tissue models. Recently, an on-chip single-cell-based culture system was developed. Small artificial networks of cardiomyocytes can be constructed and their spontaneous beating rhythms can be measured in terms of the effects of number of cells, configurations, and types of cells^27,28^.

Although isolated cardiomyocytes are relatively heterogeneous and their beating rhythms are inconsistent, even a pair of cardiomyocytes tend to synchronise when connected with each other. Features of an individual beating cell are currently measurable and a various configuration of a cellular network, which greatly affects cell-to-cell interactions, is artificially constructible. Therefore, examining how heterogeneity of cells and cell-to-cell interactions affect synchronisation in a small cluster of cardiomyocytes would be of considerable importance. There are two important observable quantities in a cardiomyocyte; one is its cell cycle (beating rate) and the other is its refractory period. In particular, a cardiomyocyte has a relatively long refractory period compared with that of a neuron. The difference in refractory periods among cardiomyocytes is expected to affect the behavior of synchronisation.

When cardiomyocytes are isolated, they only beat independently. However, if cardiomyocytes come into contact and interact with each other, their beating rhythms become synchronised. Researchers originally hypothesised that, in a network of cardiomyocytes, firing of one cardiomyocyte triggers induced firing of adjacent cardiomyocytes, and all of the cardiomyocytes start beating synchronously, and that the beating rate is tuned to the fastest cardiomyocyte^5^. However, recent experiments have shown that other cells are synchronised not to the fastest cell, but to the cell with the least fluctuation in beating rhythm^29^ (Supplementary Fig. S1).

The present study aimed is to investigate the community effect of cardiomyocytes in different configurations of networks constituted by cells with specified characteristics of beating rhythms. We also aimed to clarify how an assembly of cells acquires stability, one of the most important universal features in biological systems. Because preparing a cardiomyocyte with given properties in an *in vitro* experiment is relatively difficult, we developed a mathematical model, which explained this behavior of cardiomyocytes with high reliability. The model that we adopted in this study is a modification of the well-known integrate-and-fire model, which has been widely used as a spiking neuron model^30,31^. Using this model, we investigated the dependence of fluctuation in beating rhythm on the number of cells and that on configuration of the networks.

## Methods

We constructed a theoretical modelling for synchronisation of cardiomyocytes by modifying the integrate-and-fire model. The integrate-and-fire model disregards biological details and only focusses on causal relationships in the phenomenon. This model consists of biological oscillators or, equivalently, phase variables just as those in phase equations, but the interaction between them is instantaneous and spiky. Based on the simple Peskin’s model^32^, we included refractory periods, stochastic process, and weak cell-to-cell interactions, which modulate phase variables^19,20^. An important point is that the stochastic process and the cell-to-cell interaction are correlated through the fluctuation-dissipation theorem that gives the relation between fluctuations and linear response to external force^33,34^. Despite the simplicity of our model, it accurately reproduces most experimental results for interaction between two cardiomyocytes, though it has only one free parameter and the other parameters are determined by experiments. In the current study, we used data for 14 pairs of cardiomyocytes reported from a previous publication^29^. In the experiment^29^, cardiomyocytes that were dissociated from 13 to 14-day-old mouse embryos were used. We used Fortran and Mathematica to perform numerical simulations and to analyze the obtained data.

### Mathematical modelling

When considering a network of *N* cardiomyocytes, the model was described by the phase variables ${\varphi }_{i}(t)$ ($0\le {\varphi }_{i}(t)\le 2\pi$, $i=\mathrm{1,}\,\mathrm{2,}\ldots ,N$), which denote the state of *i*th cardiomyocytes. We assumed that the *i*th cardiomyocyte fires (beats) when ${\varphi }_{i}(t)=\mathrm{0(}\equiv 2\pi )$. This firing occurs either at ${\varphi }_{i}(t)$, reaches 2*π*, or the following conditions are satisfied: ${\varphi }_{i}(t-\mathrm{0)}\ge {\theta }_{i}\,({\varphi }_{i}(t-\mathrm{0)}:={\mathrm{lim}}_{\varepsilon \to +0}{\varphi }_{i}(t-\varepsilon ))$. Additionally, one of the cardiomyocytes connected to *i*th cardiomyocyte (e.g., *j*th cardiomyocyte) fired at a delayed time *τ* ago (i.e., ${\varphi }_{j}(t-\tau )=0$). Otherwise, we assumed that ${\varphi }_{i}(t)$ is governed by the following interacting stochastic differential equation. Our mathematical modelling for cell-*i* is as follows:

1 $$\{\begin{array}{ll}d{\varphi }_{i}(t)={\omega }_{i}dt+dW({\sigma }_{i})+{\sigma }_{i}^{2}\sum _{j}V({\varphi }_{i},{\varphi }_{j})dt & ({\varphi }_{i}(t-\mathrm{0)} < {\theta }_{i}\,{\rm{or}}\,{\varphi }_{j}(t-\tau )\ne \mathrm{0),}\\ {\varphi }_{i}(t)=0 & ({\theta }_{i}\le {\varphi }_{i}(t-\mathrm{0)}\,{\rm{and}}\,{\varphi }_{j}(t-\tau )=\mathrm{0),}\end{array}$$

where *ω*_*i*_ is the average phase velocity of cell-*i*, *θ*_*i*_ is a phase corresponding to the refractory period of cell-$i\mathrm{(0} < {\theta }_{i} < 2\pi )$, *dW*(*σ*) is a stochastic process with standard deviation *σ*, and Σ_*j*_ denotes the summation over all the cardiomyocytes connected with the *i*th cardiomyocyte. $V({\varphi }_{i},{\varphi }_{j})$ denotes the weak interaction through the membrane potential, which we assumed as the following form:

2 $$V({\varphi }_{i},{\varphi }_{j}):=\mu \,\sin ({\varphi }_{j}-{\varphi }_{i}\mathrm{).}$$

**Figure 1:**
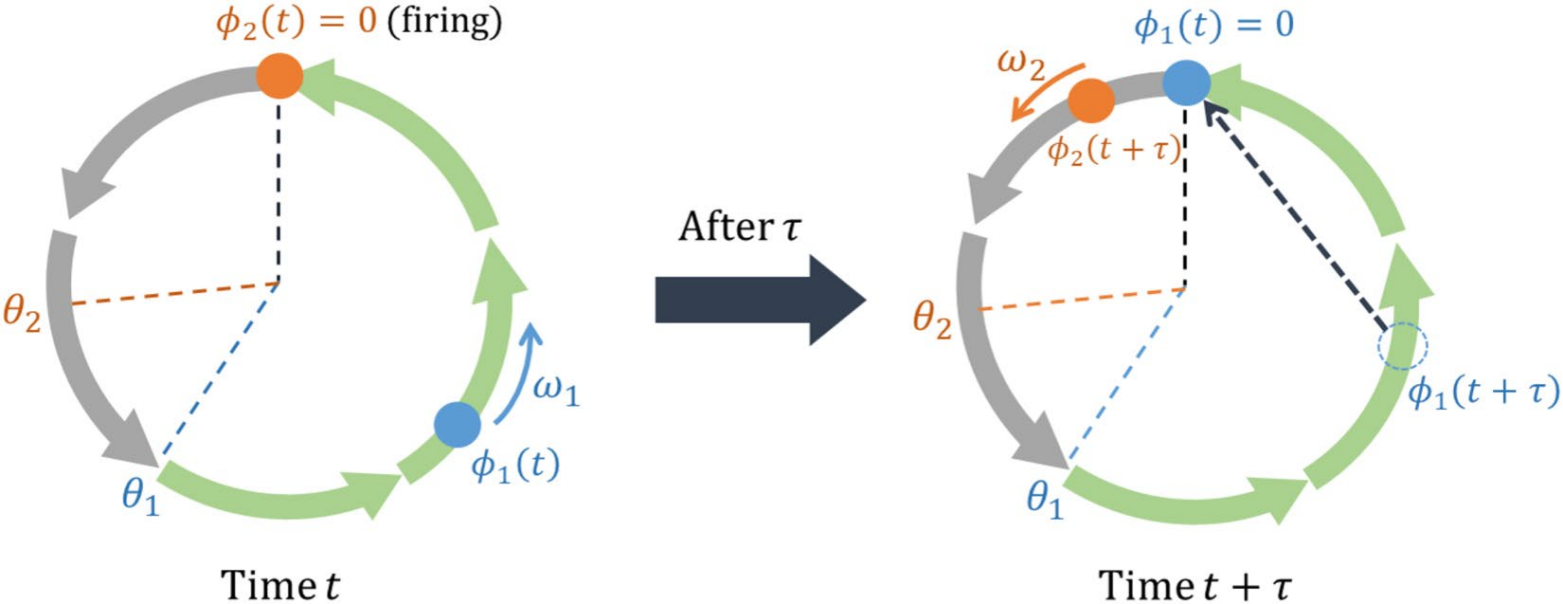
Schematic diagram of the trajectory of the state variables ${\varphi }_{i}(t)(i=\mathrm{1,2)}$. The circle represents the trajectory of a state variable in the phase space of cardiomyocytes from one firing to the next firing. If cell-2 fires at a time *t* (${\varphi }_{2}(t)$ = 0) and cell-1 is not in the refractory period, then cell-1 fires at the retardation time *τ* after cell-2 fires.

Here *μ* is a positive constant. Note that *ω*_*i*_, *θ*_*i*_, *σ*_*i*_ can be determined by single-cell experiments for each cardiomyocyte. We used an extended random walk as the stochastic process $W(\sigma )$. The positive constant *μ* is the only parameter in our model that cannot be directly measured by experiments. The dynamics of the state variable ${\varphi }_{i}(t)$ is schematically shown in Fig. 1.

### Numerical simulation method

In our simulation, the stochastic process is expressed by an extended random walk and the standard deviation is defined by $\sigma :={\rm{\Delta }}x/\sqrt{2{\rm{\Delta }}t}$, where Δ*t* and Δ*x* are the time interval and the one-step distance in the random walk, respectively. We used the following difference equations as a numerical approximation of equation (1). For almost all cardiomyocytes, we considered an ordinary random walk as follows:

3 $$\{\begin{array}{ll}{\varphi }_{i}(t+{\rm{\Delta }}t)={\varphi }_{i}(t)+{\omega }_{i}{\rm{\Delta }}t+{\rm{\Delta }}{\varphi }_{i}+{\sigma }_{i}^{2}\sum _{j}V({\varphi }_{i},{\varphi }_{j}){\rm{\Delta }}t & ({\varphi }_{i}(t) < {\theta }_{i}\,{\rm{or}}\,{\varphi }_{j}(t-\tau )\ne \mathrm{0),}\\ {\varphi }_{i}(t+{\rm{\Delta }}t)=0 & ({\theta }_{i}\le {\varphi }_{i}(t)\,{\rm{and}}\,{\varphi }_{j}(t-\tau )=\mathrm{0),}\end{array}$$

4 $$\begin{array}{ccc}{\rm{\Delta }}{\varphi }_{i} & = & \{\begin{array}{cc}+{\rm{\Delta }}{x}_{i} & (\mathrm{with}\,{\rm{probability}}\,{\rm{0}}\mathrm{.5}),\\ -{\rm{\Delta }}{x}_{i} & (\mathrm{with}\,{\rm{probability}}\,{\rm{0}}\mathrm{.5}),\end{array}\end{array}$$

where Δ*t* is the time difference interval for numerical simulations, the retardation time *τ* is set as Δ*t* × *m* nonnegative integer, and ${\rm{\Delta }}{x}_{i}=\sqrt{2{\rm{\Delta }}t\,{\sigma }_{i}^{2}}$ is the spatial difference determined from *σ*_*i*_. However, for cardiomyocytes with a large fluctuation, we could not reproduce the same fluctuation in beating rhythm by an ordinary random walk. This is because the coefficient variation (CV%), which was defined by 100× standard deviation/mean beating rate, could be less than $100\sqrt{2/3}\simeq 81.65$ ^35^. However, some cardiomyocytes have the CV% which exceed this value. Therefore, we adopted the following extended random walk, which is a history-dependent stochastic process, when beating fluctuation was larger than 81.65:

5 $$\{\begin{array}{ll}{\varphi }_{i}(t+{\rm{\Delta }}t)={\varphi }_{i}(t)+{\omega }_{i}{\rm{\Delta }}t+{\rm{\Delta }}{\tilde{\varphi }}_{i}(t)+{\sigma }_{i}^{2}\sum _{j}V({\varphi }_{i}(t),{\varphi }_{j}(t)){\rm{\Delta }}t & ({\varphi }_{i}(t) < {\theta }_{i}\,{\rm{or}}\,{\varphi }_{j}(t-\tau )\ne \mathrm{0),}\\ {\varphi }_{i}(t+{\rm{\Delta }}t)=0 & ({\theta }_{i}\le {\varphi }_{i}(t)\,{\rm{and}}\,{\varphi }_{j}(t-\tau )=\mathrm{0).}\end{array}$$

The noise term ${\rm{\Delta }}{\tilde{\varphi }}_{i}(t)$ is defined as:

6 $$\begin{array}{ccc}{\rm{\Delta }}{\tilde{\varphi }}_{i}(t) & = & \{\begin{array}{ll}+{\rm{\Delta }}{x}_{i} & ({\rm{if}}\,{\rm{\Delta }}{\tilde{\varphi }}_{i}(t-{\rm{\Delta }}t)={\rm{\Delta }}{x}_{i},{\rm{then}}\,{\rm{with}}\,{\rm{probability}}\,q),\\ 0 & (\,{\rm{if}}\,{\rm{\Delta }}{\tilde{\varphi }}_{i}(t-{\rm{\Delta }}t)={\rm{\Delta }}{x}_{i},\,{\rm{then}}\,{\rm{with}}\,{\rm{probability}}\,1-q),\\ 0 & ({\rm{\Delta }}{\tilde{\varphi }}_{i}(t-{\rm{\Delta }}t)=\mathrm{0,}\,{\rm{then}}\,{\rm{with}}\,{\rm{probability}}\,r),\\ +{\rm{\Delta }}{x}_{i} & ({\rm{\Delta }}{\tilde{\varphi }}_{i}(t-{\rm{\Delta }}t)={\rm{0}},\,{\rm{then}}\,{\rm{with}}\,{\rm{probability}}\,1-r\mathrm{).}\end{array}\end{array}$$

However,

7 $$\begin{array}{ccc}{\rm{\Delta }}{\tilde{\varphi }}_{i}\mathrm{(0)} & = & \,\{\begin{array}{cc}+{\rm{\Delta }}{x}_{i} & ({\rm{with}}\,{\rm{probability}}\,\mathrm{0.5),}\\ 0 & ({\rm{with}}\,{\rm{probability}}\,\mathrm{0.5).}\end{array}\end{array}$$

By choosing appropriate values of *q* and *r*, we could reproduce the large fluctuation observed in the experiments.

## Results

### Comparison of the model with experimental results of two cardiomyocytes

**Figure 2:**
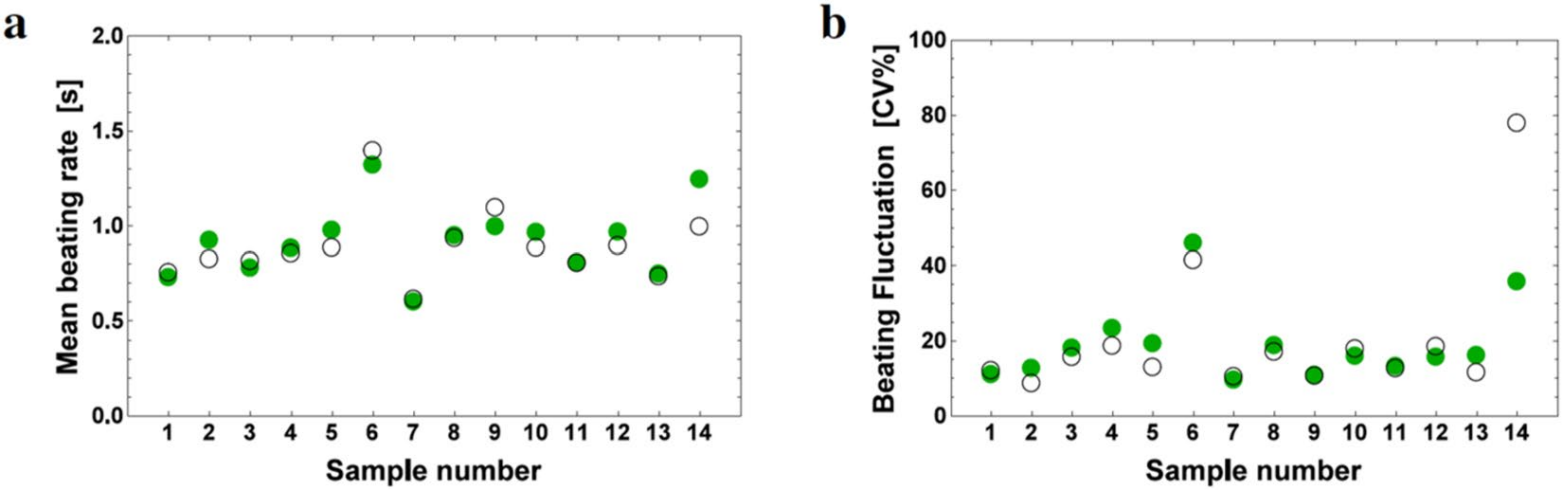
The mean beating rate and beating fluctuation after synchronisation. Numerical simulations for the 14 pairs of caridiomyocytes (28 cardiomyocytes) before synchronisation in the experiments reported previously^29^ were performed using our integrate-and -fire model. Experimental values (circles) and theoretical values (filled circles) are plotted for (**a**) the mean beating rate and (**b**) beating fluctuation (CV%). For all numerical simulations, we used the same parameter values *τ* = 0 and *μ* = 6.5. The fluctuation in beating rhythm is expressed by the CV. Mean beating rate and fluctuation for the 14 pairs after synchronisation are also shown in the Supplementary Information (Supplementary Table S2).

In the previous experiments using cultured cardiomyocytes^29^, the mean beating rate and its fluctuation before and after synchronisation were observed for 14 pairs of cardiomyocytes. We applied our mathematical model of the present study to determine whether it could reproduce the results of these pairs of cardiomyocytes. We numbered these 14 pairs from Nos1 to 14 and distinguished the two cardiomyocytes in a pair by denoting “cell-1” and “cell-2”. For each pair, we defined ${\omega }_{i},{\sigma }_{i},{\theta }_{i}$ in equation (1) for cell-*i* (*i* = 1, 2), so that the model reproduced the same mean beating rate and fluctuation in beating rhythm. Refractory periods of cardiomyocytes are almost the same as those for normal cells. Therefore, we assumed that each cell had the common refractory period *t*_*ref*_ = 0.3(s). Therefore, *θ*_*i*_ is given by ${\theta }_{i}={t}_{ref}{\omega }_{i}$. The exact values of parameters ${\omega }_{i},{\sigma }_{i},{\theta }_{i}$ $(i=\mathrm{1,}\,2)$ are shown in data tables in the Supplementary Information (Supplementary Table S1). Figure 2 shows the mean beating rates and fluctuation in the beating rhythm after synchronisation for the 14 pairs obtained by the experiments and numerical simulation by our model. We used the retardation time $\tau =0$ because it was estimated as 10^−3^~10^−4^ of the mean beating rate. We used *μ* = 6.5 in numerical simulations. By defining an evaluation function, we were able to determine the free parameter *μ* to minimize the function. The results of the numerical simulations were almost constants for a relatively wide range of *μ*. This finding indicated that our model was robust against the free parameter *μ*. The dependence of theoretical calculation on *μ* is shown in the Supplementary Information (Supplementary Note S1 and Supplementary Fig. S2). Except for pair No. 14, the simulated values accurately agree with the experimental values. Fluctuation in beating of a pair of synchronised cardiomyocytes almost coincided with that of less fluctuating cardiomyocytes, while the mean beating rate after synchronisation was widely distributed. Some synchronised cardiomyocytes coincided with faster rates, some with slower rates, and others with intermediate rates. The experimental result of pair No.14 is exceptional because it is the only pair in which fluctuation increased after synchronisation.

### Comparison with the Kuramoto model

For two coupled oscillators ${\{{\bar{\varphi }}_{i}\}}_{i=\mathrm{1,2}}$, we introduced the reaction ${A}_{i,j}f({\bar{\varphi }}_{j},{\bar{\varphi }}_{i})$, where *A*_*i*,*j*_ are constants and *f* is a function satisfying $f({x}_{1},{x}_{2})=f({x}_{1}-{x}_{2})$. For simplicity, we assumed that *f*(*x*) is 1-periodic function with *f*(−*x*) = −*f*(*x*) (e.x. *f*(*x*) = sin(*x*)) and *A*_*i*,*j*_ ≥ 0. The two-oscillators phase model (Kuramoto model) with noise is as follows: for *i*, *j* = 1, 2, *i* ≠ *j*,

8 $$d{\bar{\varphi }}_{i}(t)={\bar{\omega }}_{i}dt+{A}_{i,j}f({\bar{\varphi }}_{j}-{\bar{\varphi }}_{i})dt+{\bar{\sigma }}_{i}d{W}_{i}(t),$$

9 $${\bar{\varphi }}_{i}\mathrm{(0)}=\mathrm{0,}$$

where ${\bar{\omega }}_{i}$ and ${\bar{\sigma }}_{i}$ denote the drift and noise strength constants, respectively, and ${\{{W}_{i}\}}_{i=\mathrm{1,2}}$ is independent standard Brownian motion. For two cases (Case (i) and Case (ii)), we applied the Kuramoto model (8) and our model (1) to synchronisation of two coupled cardiomyocytes. The numerical simulation results were compared with biological experiment data^29^.

#### Case (i) A case of synchronisation to a cardiomyocyte with a fast and stable beating rhythm

**Figure 3:**
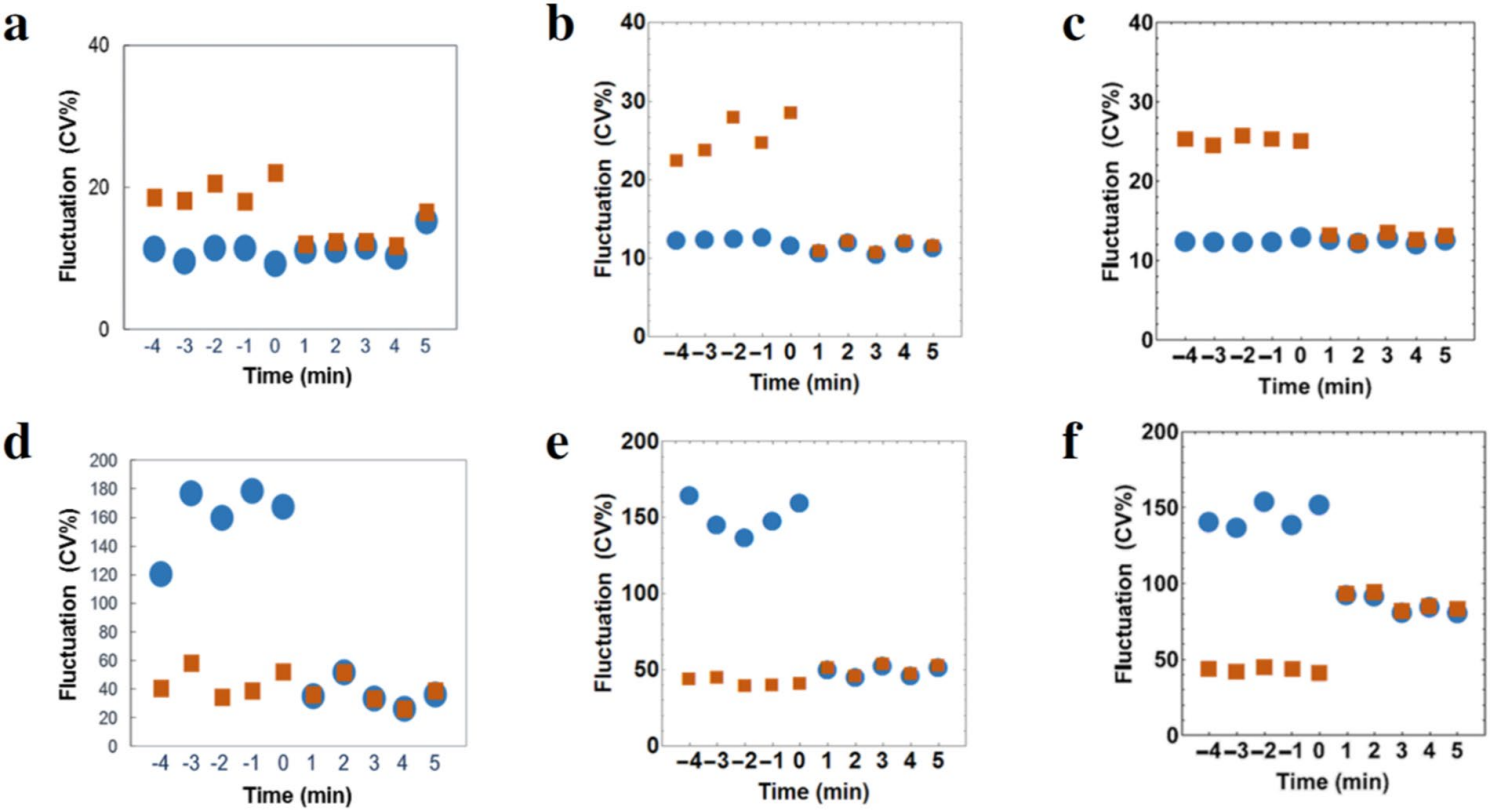
Comparison of experimental data and the two models. The change in beating fluctuation before and after synchronisation is shown. The blue circles and brown squares represent the corresponding mean values for 1 min of beating fluctuation of cell-1 and cell-2, respectively. Panels a–c show the results for Case (i), which was a case of synchronisation to a cardiomyocyte with a fast and stable beating rhythm. (**a**) The experimental result, (**b**) the numerical result of our model, and (**c**) the numerical result of the Kuramoto model with (${\bar{\omega }}_{1},{\bar{\sigma }}_{1}$) = (9.80, 0.94), (${\bar{\omega }}_{2},{\bar{\sigma }}_{2}$) = (5.09, 1.45). Panels d–f show the results for Case (ii), which was a case of synchronisation to a cardiomyocyte with a slow and stable beating rhythm. (**d**) The experimental result, (**e**) the numerical result of our model, (**f**) the numerical result of the Kuramoto model with (${\bar{\omega }}_{1},{\bar{\sigma }}_{1}$) = (5.03, 6.28), (${\bar{\omega }}_{2},{\bar{\sigma }}_{2}$) = (4.46, 1.57).

When cell-1 which had a mean beating rhythm of 0.64 s and fluctuation of 12.3 [CV%] and cell-2 with 1.23 s and 25.1 [CV%] were coupled, we found that the fast and stable cardiomyocyte (cell-1) acted as a pacemaker and the beating rhythm after synchronisation was tuned to cell-1 (Fig. 3a). We investigated whether our model and the Kuramoto model could reproduce the experimental results. Figure 3b and c shows the theoretical predictions from our model and the Kuramoto model, respectively, and both models reproduced the observations. The mean beating rate and beating fluctuation for the experimental result, our model, and the Kuramoto model are shown in the Supplementary Information (Supplementary Table S3).

#### Case (ii) A case of synchronisation to a cardiomyocyte with a slow and stable beating rhythm

When cell-1 which had a mean beating rhythm of 1.10 s and fluctuation of 149 [CV%] and cell-2 with 1.40 s and 41.2 [CV%] were coupled, we found that the slow and stable cardiomyocyte (cell-2) acted as a pacemaker and the beating rhythm after synchronisation was tuned to cell-2 (Fig. 3d). When we compared the numerical result of our model with that of the Kuramoto model, our model was closer to the experimental data than the Kuramoto model. Our model showed that the beating rhythm after synchronisation was tuned to the slow and stable cardiomyocyte (Fig. 3e). However, the Kuramoto model showed that beating fluctuation of the slow and stable cardiomyocyte was increased after synchronisation, which differed from the observations (Fig. 3f). The mean beating rate and beating fluctuation of the experimental result, those of our model and those of the Kuramoto model are shown in the Supplementary Information (Supplementary Table S4).

Therefore, our model showed that even though the mean beating rate of a cardiomyocyte was slow, a cardiomyocyte with more stable beating fluctuation dominated the beating rhythm after synchronisation. If CV values were set to a similar values, we can also obtain the same results (Supplementary Note S2, Supplementary Fig. S3 and Supplementary Table S9). In previous numerical simulations, we did not consider the effect of retardation time (*τ* = 0). When we incorporated this effect, the behavior of our model barely changed because of the existence of a refractory period much longer than *τ*. However, if the refractory period is not taken into account, then a couple of cardiomyocytes continuously fire with the period of the retardation time, which is biologically unacceptable. In a system of two cardiomyocytes, we can use a retardation time *τ* = 0, but we should consider the effect of retardation time as the system size increases. In this case, the existence of the refractory period will have significant effects on the system.

As an application of our mathematical modelling, we then performed two numerical experiments on networks of cardiomyocytes and investigated the community effect of cardiomyocytes.

### Size and configuration dependence on fluctuation of the system

**Figure 4:**
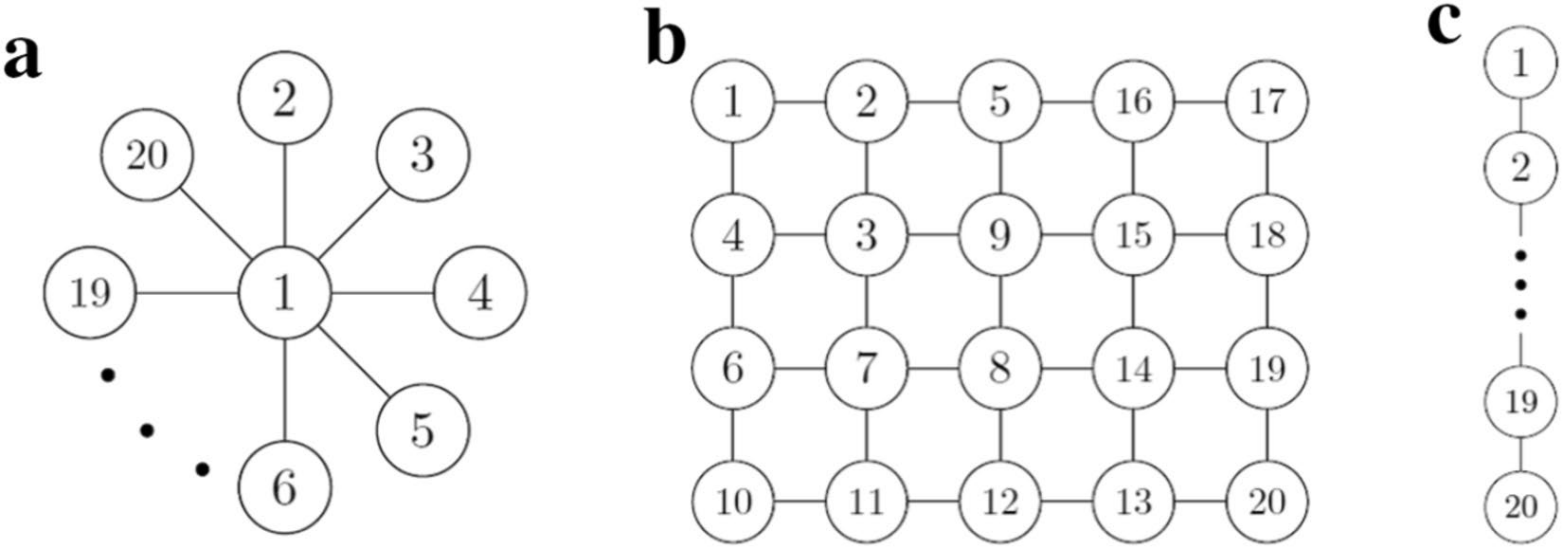
Configurations and the order of placing cells. Three types of networks of configuration are shown. (**a**) Star network, (**b**) 2D lattice network, and (**c**) 1D lattice network. A cardiomyocyte is represented as a circle and it interacts with another cardiomyocyte if they are connected by a line. Cardiomyocytes are connected in ascending order according to the numbers in the circles from 1 to 20.

First, we investigated the dependence of fluctuation in beating rhythm of cardiomyocytes on the size and configuration of the system. The configurations that we considered were star, 2D lattice and 1D lattice networks (Fig. 4).

**Figure 5:**
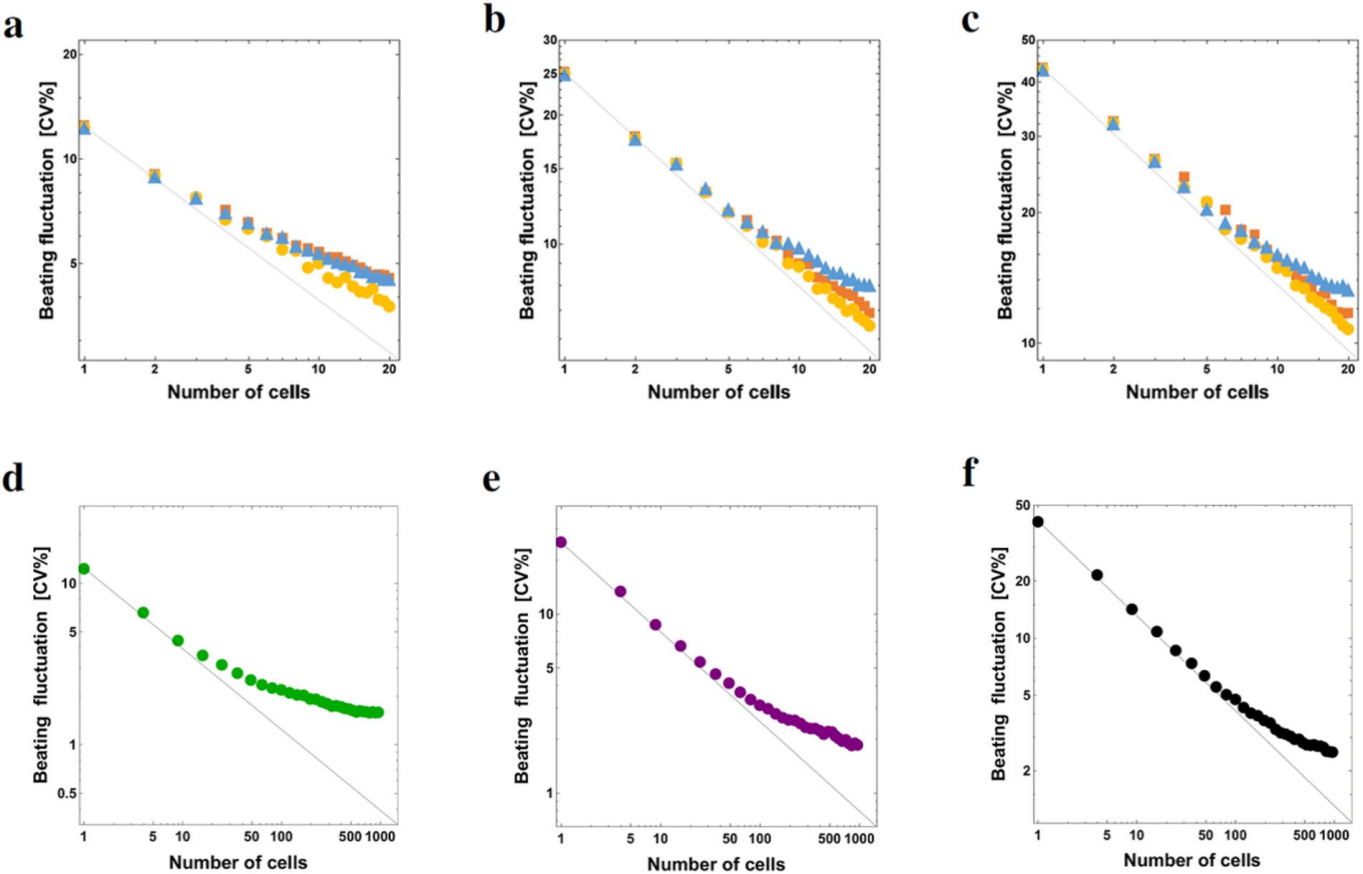
Size dependence of fluctuation for three types of configuration and for a large network. Size dependence of fluctuation is shown in double logarithmic graphs. The components of the network are model cardiomyocytes with the same characteristics. Panels a–c show the size dependence of fluctuation for three types of configurations: (**a**) $\omega =\mathrm{9.80,}\,\sigma =\mathrm{0.69,}\,\theta =2.94$, (**b**) $\omega =\mathrm{5.00,}\,\sigma =\mathrm{1.01,}\,\theta =1.50$ and (**c**) $\omega =\mathrm{2.10,}\,\sigma =\mathrm{1.18,}\,\theta =0.63$. Brown squares indicate beating fluctuation (CV%) of cardiomyocytes in the star network, orange circles indicate beating fluctuation in the 2D lattice network, and blue triangles indicate beating fluctuation in the 1D lattice network. Panels d–f show the size dependence of fluctuation for a larger 2D lattice network. (**d**) $\omega =\mathrm{9.80,}\,\sigma =\mathrm{0.69,}\,\theta =2.94$, (**e**) $\omega =\mathrm{5.00,}\,\sigma =\mathrm{1.01,}\,\theta =1.50$, and (**f**) $\omega =\mathrm{2.10,}\,\sigma =\mathrm{1.18,}\,\theta =0.63$. The black straight line denotes ∝*N* ^−1/2^ where *N* is the number of cardiomyocytes in the network.

Figures 5a–c shows the size dependence of fluctuation of networks with three types of configurations where all the elements have the same beating properties. The cardiomyocyte that was used in Fig. 5a was cell-1 of pair No.1, which had a mean beating rhythm of 0.64 s and fluctuation of 12.4 [CV%], that in Fig. 5b was cell-2 of pair No.1 with 1.23 s and 25.1 [CV%], and that in Fig. 5c was cell-2 of pair No.8 with 2.71 s and 43.0 [CV%]. In all configurations, fluctuation rapidly decreased with an increase in the size of the system. Among the three configurations, a reduction in fluctuation was most rapid in the 2D lattice network, and fluctuation in the 1D lattice network was always larger than that in the other two configurations. Furthermore, we considered the larger size (about 1000 cells) of the network in the 2D lattice network. Figure 5d–f shows the size dependence of fluctuation of the 2D lattice network. Similar to Fig. 5a–c, all the elements had the same beating properties. In all cases, the beating fluctuation decreased as the community size increased. In Fig. 5(a–f), beating fluctuation of model cardiomyocytes are much different, but if CV values were set to a similar values, the same results was obtained (Supplementary Figs S4 and S5). For an ordinary stochastic ensemble, such as an independently identical distributed ensemble, the dependence of standard deviation of fluctuation on system size *N* was proportional to *N* ^−1/2^. However, the data of fluctuation plotted on the graph (Fig. 5) considerably deviated from the line of *N* ^−1/2^ and the feature of beating fluctuation was relatively different from that of ordinary stochastic ensembles.

### Dependence of cellular properties and numbers on fluctuation of the system

**Figure 6:**
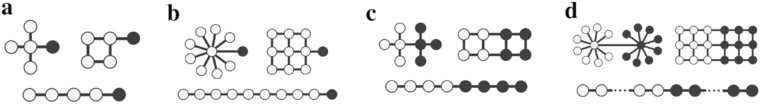
Configurations of a combination between a referential network and a single cell or an assembly of cells. In panels a and b, the filled circles denote a single cell, which adds to the referential network, of which cells are denoted by open circles. (**a**) Referential network of four cells + a single cell and (**b**) that of nine cells + a single cell. Panels c and d show the configurations of combined subsystems of four cells and those of nine cells. The cells in referential networks are denoted by open circles and those in counterparts are denoted by filled circles. (**c**) Referential network of four cells + four cells and (**d**) that of nine cells + nine cells.

We then investigated the change in beating rhythms after connecting two subsystems of cardiomyocytes. First we composed referential subsystems of four model cells and nine model cells so that these subsystems had the property of a standard beating rhythm (mean beating rate 1.20~1.30 s; fluctuation 15.0~20.0 [CV%]) As for the subsystems which are connected to referential subsystems, we considered subsystems consisting of four types of cardiomyocytes: (i) first and stable cell, (ii) first and unstable cell, (iii) slow and stable cell, and (iv) slow and unstable cell. For networks, we considered the three types of configurations shown in Fig. 4. A single cell was connected to a centre cell of the referential star network, to a cell on a link of the referential 2D lattice network, and to a cell on an edge of the 1D lattice network (Fig. 6a,b). In case of subsystems of the same size, we connected them by a single link between two cells at the same positions in the configuration. We used the two centre cells for the star networks, cells on the links for the 2D lattice network, and the two cells at the edges for the 1D lattice network (Fig. 6c,d).

**Figure 7:**
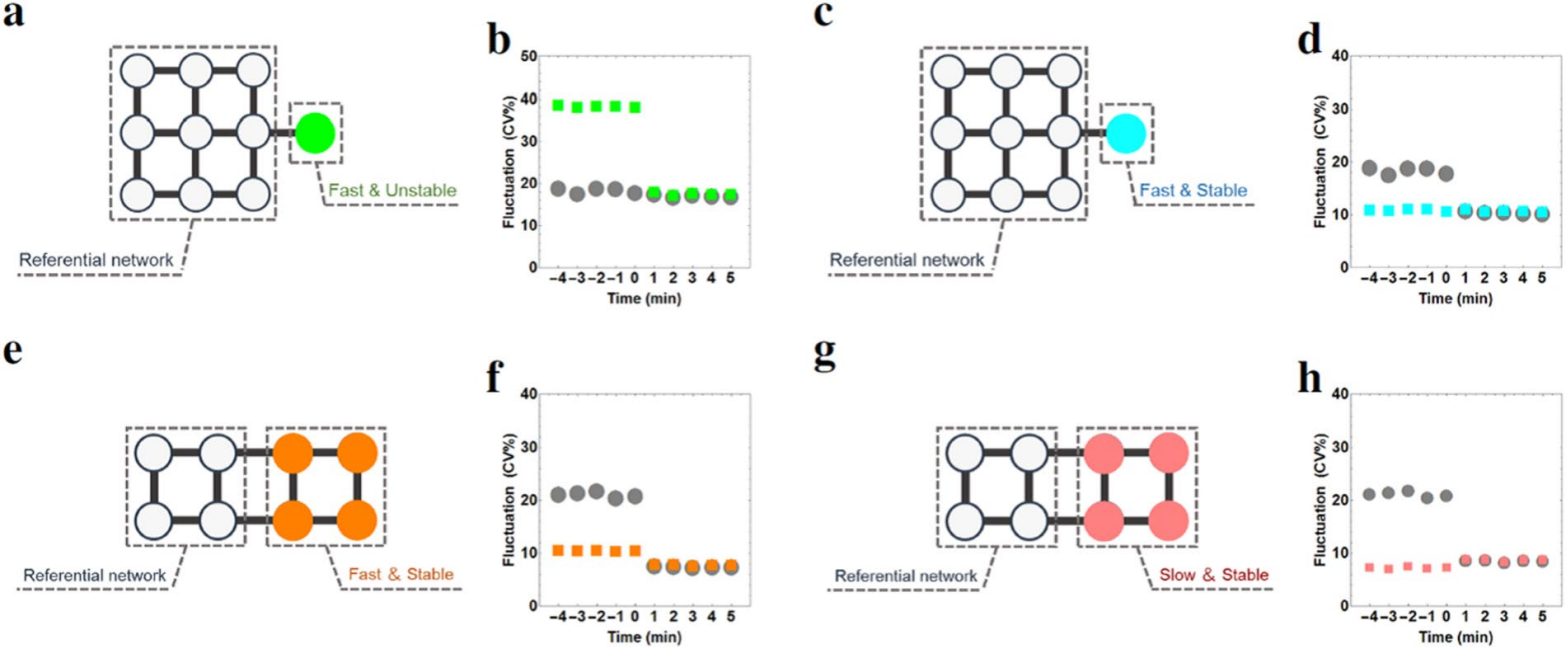
Change in the beating fluctuation before and after synchronisation. (**a**) The referential subsystem is the 2D lattice network and the counterpart is the single cell with a fast and unstable beating rhythm. (**b**) The change in mean value of beating fluctuation. The data for the referential networks and the counterparts are shown by circles and squares, respectively. The circles and squares show the corresponding mean values for 1 min of beating fluctuation. The results for the other combined systems (**c**,**e**,**g**) are shown similarly in (**d**,**f**,**h**), respectively.

The results are shown in the Supplementary Information (Supplementary Tables S5–S8). The mean beating rate and fluctuation (CV%) of each cell were estimated by the data of 3,000 firings. We showed three typical results of the numerical simulation. First, we considered the referential 2D lattice network with nine cells and a single cell that had a fast and unstable beating rhythm (Fig. 7a). In this case, the beating rhythm of the system after connection was tuned to the rhythm of nine cells with more stable beating (Fig. 7b). Second, the rhythm of a single cell was fast and stable (Fig. 7c). We then found that even a single cell could lower fluctuation of the referential network and the synchronised beating rhythm was tuned to a stable single cell (Fig. 7d). Finally, we considered a referential 2D lattice network with four cells and four cells grouped with a fast and stable beating rhythm (Fig. 7e). The beating rhythm after connection was also tuned to the rhythm of the more stable group (Fig. 7f). In the above three cases (Fig. 7a–f), every cell started synchronising after connection and fluctuation of the cells became equal in the combined system. However, in the case of 1D lattice network in Fig. 6d, the cells near the edge of 1D lattice network with nine cells showed an exceptionally large fluctuation compared with the other cells. Synchronisation did not occur in the referential 1D lattice network with nine cells + nine cells with a fast and stable beating rhythm. Furthermore, fluctuations of combined systems reduced their intensity, except for when there was a single cell or cell group with a slow and stable property. However, the increment in fluctuation was small, even in these cases (e.g., Fig. 7g,h). When a referential subsystem was connected to a counterpart consisting of one of the other three types of cells, the constituent cells acquired a common intensity of fluctuation. The intensity was intermediate between that of the prior two subsystems, but was similar to that of the less fluctuating subsystem.

## Discussion

**Figure 8:**
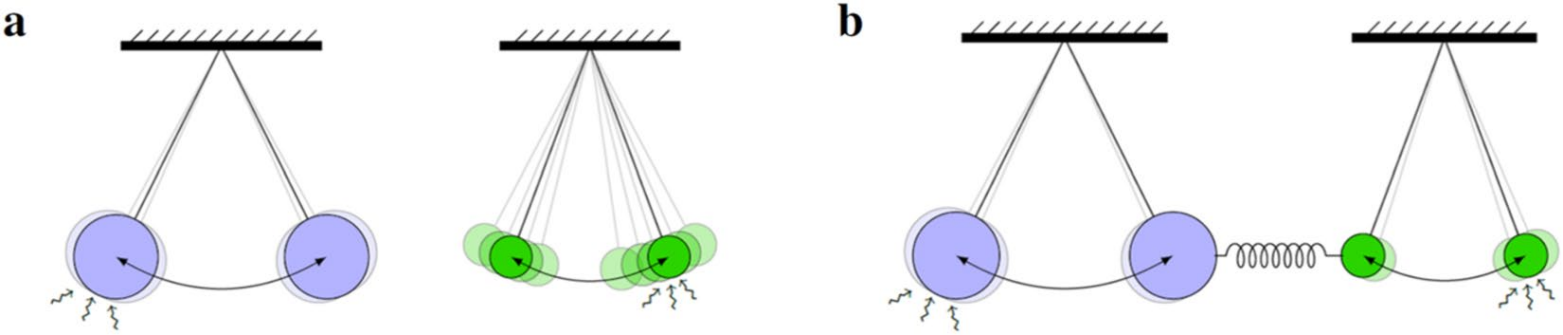
Schematic explanation of why the beating rhythm tend to be synchronised to that of more stable cardiomyocytes after connection of two cardiomyocytes. A stable cardiomyocyte can be compared with a heavy pendulum and an unstable cardiomyocyte with a light pendulum. (**a**) External fluctuation has little effect on a pendulum’s period of swing if its weight is heavy, but has strong effects if its weight is light. (**b**) When two pendulums are coupled and synchronised, their period of swing is close to that of the heavier pendulum, and fluctuation will be reduced because the total mass of weight increases.

We investigated the community effect of networks of cardiomyocytes by using an interacting integrate-and-fire model with a refractory period. The reliability of the present model was verified by accurately reproducing recent experiments on pairs of cultured cardiomyocytes by Kojima *et al*.^29^, despite the fact that the model has only one tuning parameter. Interestingly, the fluctuation observed in their experiments cannot be explained by simple Brownian motion or equivalently random walks. This is because some of the fluctuations in beating rhythm (CV%) exceeded the theoretical limit evaluated for Brownian motion. An important observation in their experiments is the finding that a pair of cardiomyocytes, when connected, tended to beat synchronously at a rate of the cell with a stable beating rhythm, but not the cell with a faster beating rhythm. This community effect of cardiomyocytes towards stability was confirmed with the present model by examining networks of various configurations and constituent cells with various beating rhythms. Even a single stable cardiomyocyte could lower fluctuation of a network consisting of some cardiomyocytes. The reason why an unstable cardiomyocyte tends to follow a stable cardiomyocyte may be explained as follows. A stable cardiomyocyte has the property where its dynamics are only slightly affected by external or internal disturbance. Therefore, there is little effect of interactions from neighboring cardiomyocytes, while an unstable cardiomyocyte has the opposite property, and is strongly affected by its neighbors. A stable cardiomyocyte corresponds to a pendulum with a heavy mass in contrast to an unstable cardiomyocyte that corresponds to that with a light mass (Fig. 8). When we connect these pendulums, clearly the pendulum with a light mass tends to follow that with a heavy mass. This feature is a consequence of the fluctuation-dissipation theorem, which provides a universal relation between fluctuation and a linear response^33,36^. In our model, the factor ${\sigma }_{i}^{2}$ of the interaction term ${\sum }_{j}V({\varphi }_{j},{\varphi }_{i})$ in equation (1) was due to this theorem, and it plays an essential role in stabilising the beating rhythm after synchronisation. Stability is one of the most important and universal features of biological systems. Interestingly, one of the origins of biological stability is a universal principle in statistical physics, that is, fluctuation-dissipation theorem.

## Electronic supplementary material

Supplementary Information
