# Supplementary material for: Community effect of cardiomyocytes in beating rhythms is determined by stable cells
Source: Sci Rep. 2017 Nov 13;7:15450. doi: 10.1038/s41598-017-15727-5 (PMC5684290; doi:10.1038/s41598-017-15727-5)
Supplement: Supplementary file 1 — Supplementary Information [file 41598_2017_15727_MOESM1_ESM.pdf]

Supplementary information

Community effect of cardiomyocytes in beating rhythms is determined by stable cells

Tatsuya Hayashi[1,2], Tetsuji Tokihiro[1,2*], Hiroki Kurihara[2,3] and Kenji Yasuda[2,4*]

[1] Graduate School of Mathematical Sciences, the University of Tokyo, 3-8-1 Komaba, Tokyo 153-8941, Japan,

[2] CREST, Japan Science and Technology Agency, 4-1-8 Honcho, Kawaguchi, Saitama 332-0012, Japan,

[3] Graduate School of Medicine and Faculty of Medicine, the University of Tokyo, 7-3-1 Hongo, Tokyo 113-0033, Japan,

[4] Faculty of Science and Engineering, Waseda University, 3-4-1 Okubo, Tokyo 169-8555, Japan,

[*] Correspondence and requests for materials should be addressed to T.T. or K.Y.
(email: toki@ms.u-tokyo.ac.jp, yasuda@waseda.jp)

Supplementary Table S1: The values of parameters ω_i, σ_i, θ_i $(i = 1, 2)$ for 14 pairs.

	Cell-1			Cell-2		
No.	ω_1	σ_1	θ_1	ω_2	σ_2	θ_2
1	9.80	0.69	2.94	5.00	1.01	1.50
2	6.80	0.76	2.04	6.20	0.93	1.86
3	8.40	1.02	2.52	5.01	1.99	1.50
4	7.20	0.94	2.16	1.50	2.48	0.45
5	6.70	0.68	2.01	0.50	1.91	0.15
6	1.40	3.54	0.42	4.30	1.55	1.29
7	10.60	0.95	3.18	9.90	0.64	2.97
8	9.75	1.12	2.93	2.10	1.18	0.63
9	7.50	0.57	2.25	3.50	0.66	1.05
10	10.80	1.72	3.24	5.15	0.72	1.55
11	8.75	1.08	2.63	6.75	0.81	2.03
12	10.90	1.75	3.27	5.15	0.72	1.55
13	11.80	1.33	3.54	5.74	0.98	1.72
14	14.40	1.80	4.32	0.88	0.91	0.26

Supplementary Table S2: Comparison between experimental results and numerical simulations after synchronization. The symbol 'T' denotes the mean beating rate and 'F' the beating fluctuation.

	Experimental results		Numerical simulations			
No.	T^{ex} (s)	F^{ex} (CV %)	T_1 (s)	F_1 (CV %)	T_2 (s)	F_2 (CV %)
1	0.76	12.3	0.74	11.4	0.74	11.3
2	0.83	8.9	0.93	13.0	0.93	13.0
3	0.82	15.9	0.79	18.4	0.79	18.5
4	0.86	18.9	0.89	23.7	0.89	23.7
5	0.89	13.2	0.98	19.6	0.98	19.7
6	1.40	41.7	1.33	46.3	1.33	46.3
7	0.62	10.7	0.61	9.90	0.61	9.60
8	0.94	17.3	0.95	19.1	0.96	19.1
9	1.10	10.9	1.00	11.2	1.00	11.1
10	0.89	18.1	0.97	16.2	0.97	16.2
11	0.81	12.9	0.81	13.5	0.81	13.4
12	0.90	18.7	0.97	16.0	0.97	15.9
13	0.74	11.8	0.75	16.4	0.75	16.4
14	1.00	78.1	1.25	36.1	1.25	36.0

Supplementary Table S3: Comparison between the experimental result and the numerical results. The symbol T_i and F_i denote the mean beating rate and the beating fluctuation of the cell-$i(i = 1, 2)$, respectively. The symbol T denotes the mean beating rate and F the beating fluctuation after synchronization.

	Before synchronization				After synchronization	
	T_1(s)	F_1(CV%)	T_2(s)	F_2(CV%)	T(s)	F(CV%)
Experiments	0.64	12.3	1.23	25.1	0.76	12.3
Our model	0.64	12.3	1.23	25.1	0.74	11.4
Kuramoto model	0.64	12.3	1.23	25.1	0.85	12.7

Supplementary Table S4: Comparison between the experimental result and the numerical results. The symbol T_i and F_i denote the mean beating rate and the beating fluctuation of the cell-$i (i = 1, 2)$, respectively. The symbol T denotes the mean beating rate and F the beating fluctuation after synchronization.

	Before synchronization				After synchronization	
	T_1(s)	F_1(CV%)	T_2(s)	F_2(CV%)	T(s)	F(CV%)
Experiments	1.1	149	1.4	41.2	1.4	41.7
Our model	1.1	149	1.4	41.2	1.3	46.3
Kuramoto model	1.1	149	1.4	41.2	1.3	86.8

Supplementary Table S5: Interaction between a referential subsystem of four cells and a single cell

		**Radial like pattern**		**Lattice like pattern**		**Line like pattern**	
		cell-community	fast stable	cell-community	fast stable	cell-community	fast stable
Before connection	T (s)	1.29 1.29 1.29 1.29	0.85	1.34 1.34 1.34 1.34	0.85	1.30 1.30 1.30 1.30	0.85
	F (CV%)	20.9 20.9 20.9 20.9	11.8	21.4 21.4 21.4 21.4	11.8	21.6 21.6 21.6 21.6	11.8
After connection	T (s)	0.92 0.92 0.92 0.92	0.92	0.93 0.93 0.93 0.93	0.93	0.91 0.91 0.91 0.91	0.91
	F (CV%)	10.7 10.7 10.7 10.7	10.7	10.8 10.8 10.8 10.8	10.7	10.9 10.9 10.9 10.9	10.9
		cell-community	fast unstable	cell-community	fast unstable	cell-community	fast unstable
Before connection	T (s)	1.29 1.29 1.29 1.29	0.56	1.34 1.34 1.34 1.34	0.56	1.30 1.30 1.30 1.30	0.56
	F (CV%)	20.9 20.9 20.9 20.9	29.0	21.4 21.4 21.4 21.4	29.0	21.6 21.6 21.6 21.6	29.0
After connection	T (s)	1.01 1.01 1.01 1.01	1.01	1.02 1.02 1.02 1.02	1.02	0.99 0.99 0.99 0.99	0.99
	F (CV%)	17.7 17.7 17.7 17.7	17.8	18.2 18.2 18.2 18.2	18.3	18.6 18.6 18.6 18.6	18.7
		cell-community	slow stable	cell-community	slow stable	cell-community	slow stable
Before connection	T (s)	1.29 1.29 1.29 1.29	1.79	1.34 1.34 1.34 1.34	1.79	1.30 1.30 1.30 1.30	1.79
	F (CV%)	20.9 20.9 20.9 20.9	9.21	21.4 21.4 21.4 21.4	9.21	21.6 21.6 21.6 21.6	9.21
After connection	T (s)	1.51 1.51 1.51 1.51	1.51	1.55 1.55 1.55 1.55	1.55	1.48 1.48 1.48 1.48	1.48
	F (CV%)	11.2 11.3 11.3 11.3	11.2	11.3 11.2 11.3 11.3	11.2	13.9 13.8 13.7 13.6	13.7
		cell-community	slow unstable	cell-community	slow unstable	cell-community	slow unstable
Before connection	T (s)	1.29 1.29 1.29 1.29	2.78	1.34 1.34 1.34 1.34	2.78	1.30 1.30 1.30 1.30	2.78
	F (CV%)	20.9 20.9 20.9 20.9	43.3	21.4 21.4 21.4 21.4	43.3	21.6 21.6 21.6 21.6	43.3
After connection	T (s)	1.49 1.49 1.49 1.49	1.49	1.54 1.54 1.54 1.54	1.54	1.48 1.48 1.48 1.48	1.48
	F (CV%)	20.1 20.1 20.2 20.1	20.2	19.4 19.4 19.4 19.4	19.4	20.6 20.5 20.5 20.5	20.5

Supplementary Table S6: Interaction between a referential subsystem of nine cells and four kinds of subsystems of a single cell

		**Radial like pattern**									
		cell-community									fast stable
Before connection	T (s)	1.24	1.24	1.24	1.24	1.24	1.24	1.24	1.24	1.24	0.85
	F (CV%)	14.8	14.8	14.8	14.8	14.8	14.8	14.8	14.8	14.8	11.8
After connection	T (s)	0.96	0.96	0.96	0.96	0.96	0.96	0.96	0.96	0.96	0.96
	F (CV%)	9.92	9.92	9.94	10.0	9.93	9.91	9.93	9.92	9.93	9.97
		cell-community									fast unstable
Before connection	T (s)	1.24	1.24	1.24	1.24	1.24	1.24	1.24	1.24	1.24	0.56
	F (CV%)	14.8	14.8	14.8	14.8	14.8	14.8	14.8	14.8	14.8	29.0
After connection	T (s)	1.09	1.09	1.09	1.09	1.09	1.09	1.09	1.09	1.09	1.09
	F (CV%)	13.6	13.6	13.6	13.6	13.6	13.6	13.6	13.6	13.6	13.7
		cell-community									slow stable
Before connection	T (s)	1.24	1.24	1.24	1.24	1.24	1.24	1.24	1.24	1.24	1.79
	F (CV%)	14.8	14.8	14.8	14.8	14.8	14.8	14.8	14.8	14.8	9.21
After connection	T (s)	1.39	1.39	1.39	1.39	1.39	1.39	1.39	1.39	1.39	1.39
	F (CV%)	10.7	10.7	10.7	10.7	10.7	10.7	10.7	10.7	10.7	10.7
		cell-community									slow unstable
Before connection	T (s)	1.24	1.24	1.24	1.24	1.24	1.24	1.24	1.24	1.24	2.78
	F (CV%)	14.8	14.8	14.8	14.8	14.8	14.8	14.8	14.8	14.8	43.3
After connection	T (s)	1.33	1.33	1.33	1.33	1.33	1.33	1.33	1.33	1.33	1.33
	F (CV%)	14.5	14.5	14.5	14.5	14.5	14.5	14.5	14.5	14.5	14.5

		**Lattice like pattern**									
		cell-community									fast stable
Before connection	T (s)	1.31	1.31	1.31	1.31	1.31	1.31	1.31	1.31	1.31	0.85
	F (CV%)	14.8	14.8	14.8	14.8	14.8	14.8	14.8	14.8	14.8	11.8
After connection	T (s)	0.96	0.96	0.96	0.96	0.96	0.96	0.96	0.96	0.96	0.96
	F (CV%)	10.5	10.5	10.5	10.5	10.5	10.5	10.5	10.5	10.5	10.5
		cell-community									fast unstable
Before connection	T (s)	1.31	1.31	1.31	1.31	1.31	1.31	1.31	1.31	1.31	0.56
	F (CV%)	14.8	14.8	14.8	14.8	14.8	14.8	14.8	14.8	14.8	29.0
After connection	T (s)	1.10	1.10	1.10	1.10	1.10	1.10	1.10	1.10	1.10	1.10
	F (CV%)	14.2	14.2	14.2	14.2	14.2	14.2	14.2	14.2	14.2	14.3
		cell-community									slow stable
Before connection	T (s)	1.31	1.31	1.31	1.31	1.31	1.31	1.31	1.31	1.31	1.79
	F (CV%)	14.8	14.8	14.8	14.8	14.8	14.8	14.8	14.8	14.8	9.21
After connection	T (s)	1.45	1.45	1.45	1.45	1.45	1.45	1.45	1.45	1.45	1.45
	F (CV%)	10.4	10.3	10.3	10.3	10.3	10.4	10.3	10.3	10.3	10.3
		cell-community									slow unstable
Before connection	T (s)	1.31	1.31	1.31	1.31	1.31	1.31	1.31	1.31	1.31	2.78
	F (CV%)	14.8	14.8	14.8	14.8	14.8	14.8	14.8	14.8	14.8	43.3
After connection	T (s)	1.40	1.40	1.40	1.40	1.40	1.40	1.40	1.40	1.40	1.40
	F (CV%)	14.4	14.4	14.4	14.4	14.3	14.4	14.4	14.4	14.3	14.4

		**Line like pattern**									
		cell-community									fast stable
Before connection	T (s)	1.21	1.21	1.21	1.21	1.21	1.21	1.21	1.21	1.21	0.85
	F (CV%)	15.5	15.5	15.5	15.5	15.5	15.5	15.5	15.5	15.5	11.8
After connection	T (s)	0.92	0.91	0.91	0.91	0.91	0.91	0.91	0.91	0.91	0.91
	F (CV%)	10.6	10.5	10.5	10.5	10.5	10.5	10.5	10.5	10.5	10.5
		cell-community									fast unstable
Before connection	T (s)	1.21	1.21	1.21	1.21	1.21	1.21	1.21	1.21	1.21	0.56
	F (CV%)	15.5	15.5	15.5	15.5	15.5	15.5	15.5	15.5	15.5	29.0
After connection	T (s)	0.99	0.99	0.99	0.99	0.99	0.99	0.99	0.99	0.99	0.99
	F (CV%)	16.8	16.7	16.7	16.7	16.7	16.7	16.7	16.7	16.7	16.8
		cell-community									slow stable
Before connection	T (s)	1.21	1.21	1.21	1.21	1.21	1.21	1.21	1.21	1.21	1.79
	F (CV%)	15.5	15.5	15.5	15.5	15.5	15.5	15.5	15.5	15.5	9.21
After connection	T (s)	1.31	1.31	1.31	1.31	1.31	1.31	1.31	1.31	1.31	1.31
	F (CV%)	14.7	14.6	14.6	14.6	14.6	14.6	14.6	14.6	14.6	14.6
		cell-community									slow unstable
Before connection	T (s)	1.21	1.21	1.21	1.21	1.21	1.21	1.21	1.21	1.21	2.78
	F (CV%)	15.5	15.5	15.5	15.5	15.5	15.5	15.5	15.5	15.5	43.3
After connection	T (s)	1.29	1.29	1.29	1.29	1.29	1.29	1.29	1.29	1.29	1.29
	F (CV%)	15.6	15.6	15.5	15.5	15.5	15.5	15.5	15.5	15.5	15.5

Supplementary Table S7: Interaction between a referential subsystem of four cells and four kinds of subsystems of four cells

		**Radial like pattern**		**Lattice like pattern 1**		**Lattice like pattern 2**		**Line like pattern**	
		cell-community	fast stable	cell-community	fast stable	cell-community	fast stable	cell-community	fast stable
Before connection	T (s)	1.29 1.29 1.29 1.29	0.66 0.66 0.66 0.66	1.34 1.34 1.34 1.34	0.62 0.62 0.62 0.62	1.34 1.34 1.34 1.34	0.62 0.62 0.62 0.62	1.30 1.30 1.30 1.30	0.62 0.62 0.62 0.62
	F (CV%)	20.9 20.9 20.9 20.9	6.90 6.90 6.90 6.90	21.4 21.4 21.4 21.4	6.62 6.62 6.62 6.62	21.4 21.4 21.4 21.4	6.62 6.62 6.62 6.62	21.6 21.6 21.6 21.6	6.70 6.70 6.70 6.70
After connection	T (s)	0.64 0.64 0.64 0.64	0.64 0.64 0.64 0.64	0.64 0.64 0.64 0.64	0.64 0.64 0.64 0.64	0.66 0.66 0.66 0.66	0.66 0.66 0.66 0.66	0.63 0.63 0.63 0.63	0.63 0.63 0.63 0.63
	F (CV%)	7.98 7.38 7.96 7.73	7.13 7.22 7.23 7.22	6.69 7.32 7.05 6.69	6.71 6.74 6.74 6.78	6.69 6.69 6.69 6.68	6.68 6.75 6.68 6.77	8.37 7.19 7.19 8.12	7.17 7.10 7.23 7.38
		cell-community	fast unstable	cell-community	fast unstable	cell-community	fast unstable	cell-community	fast unstable
Before connection	T (s)	1.29 1.29 1.29 1.29	1.00 1.00 1.00 1.00	1.34 1.34 1.34 1.34	1.05 1.05 1.05 1.05	1.05 1.05 1.05 1.05	1.05 1.05 1.05 1.05	1.30 1.30 1.30 1.30	1.01 1.01 1.01 1.01
	F (CV%)	20.9 20.9 20.9 20.9	24.8 24.8 24.8 24.8	21.4 21.4 21.4 21.4	24.7 24.7 24.7 24.7	21.4 21.4 21.4 21.4	24.7 24.7 24.7 24.7	21.6 21.6 21.6 21.6	24.7 24.7 24.7 24.7
After connection	T (s)	1.14 1.14 1.14 1.14	1.14 1.14 1.14 1.14	1.19 1.19 1.19 1.19	1.19 1.19 1.19 1.19	1.22 1.22 1.22 1.22	1.22 1.22 1.22 1.22	1.14 1.14 1.14 1.14	1.14 1.14 1.14 1.14
	F (CV%)	16.9 16.9 16.9 16.9	16.9 16.9 16.9 16.9	17.0 17.0 17.0 17.0	17.0 17.0 17.0 17.1	16.9 16.9 16.9 16.9	16.9 16.9 16.9 16.9	17.9 17.9 17.9 17.9	17.9 17.9 17.9 18.0
		cell-community	slow stable	cell-community	slow stable	cell-community	slow stable	cell-community	slow stable
Before connection	T (s)	1.29 1.29 1.29 1.29	1.67 1.67 1.67 1.67	1.34 1.34 1.34 1.34	1.69 1.69 1.69 1.69	1.34 1.34 1.34 1.34	1.69 1.69 1.69 1.69	1.30 1.30 1.30 1.30	1.66 1.66 1.66 1.66
	F (CV%)	20.9 20.9 20.9 20.9	10.8 10.8 10.8 10.8	21.4 21.4 21.4 21.4	9.96 9.96 9.96 9.96	21.4 21.4 21.4 21.4	9.96 9.96 9.96 9.96	21.6 21.6 21.6 21.6	10.0 10.0 10.0 10.0
After connection	T (s)	1.46 1.46 1.46 1.46	1.46 1.46 1.46 1.46	1.50 1.50 1.50 1.50	1.50 1.50 1.50 1.50	1.56 1.56 1.56 1.56	1.56 1.56 1.56 1.56	1.43 1.43 1.43 1.43	1.43 1.43 1.43 1.43
	F (CV%)	11.5 11.6 11.6 11.5	11.5 11.5 11.5 11.5	11.9 11.8 11.9 11.9	11.8 11.8 11.8 11.8	10.3 10.2 10.2 10.2	10.2 10.2 10.2 10.2	14.2 14.1 14.1 14.1	14.1 14.1 14.1 14.1
		cell-community	slow unstable	cell-community	slow unstable	cell-community	slow unstable	cell-community	slow unstable
Before connection	T (s)	1.29 1.29 1.29 1.29	1.87 1.87 1.87 1.87	1.34 1.34 1.34 1.34	1.90 1.90 1.90 1.90	1.34 1.34 1.34 1.34	1.90 1.90 1.90 1.90	1.30 1.30 1.30 1.30	1.91 1.91 1.91 1.91
	F (CV%)	20.9 20.9 20.9 20.9	53.7 53.7 53.7 53.7	21.4 21.4 21.4 21.4	49.8 49.8 49.8 49.8	21.4 21.4 21.4 21.4	49.8 49.8 49.8 49.8	21.6 21.6 21.6 21.6	54.4 54.4 54.4 54.4
After connection	T (s)	1.29 1.29 1.29 1.29	1.29 1.29 1.29 1.29	1.34 1.34 1.34 1.34	1.34 1.34 1.34 1.34	1.39 1.39 1.39 1.39	1.39 1.39 1.39 1.39	1.27 1.27 1.27 1.27	1.27 1.27 1.27 1.27
	F (CV%)	21.3 21.3 21.3 21.3	21.3 21.3 21.3 21.3	20.3 20.3 20.3 20.3	20.3 20.3 20.3 20.3	21.3 21.3 21.3 21.3	21.3 21.3 21.3 21.3	21.3 21.3 21.3 21.3	21.3 21.3 21.3 21.3

Supplementary Table S8: Interaction between a referential subsystem of nine cells and four kinds of subsystems of nine cells

		Radial like pattern																		Lattice like pattern 1																	
		cell-community									fast stable									cell-community									fast stable								
Before connection	T (s)	1.24	1.24	1.24	1.24	1.24	1.24	1.24	1.24	1.24	0.59	0.59	0.59	0.59	0.59	0.59	0.59	0.59	0.59	1.31	1.31	1.31	1.31	1.31	1.31	1.31	1.31	1.31	0.59	0.59	0.59	0.59	0.59	0.59	0.59	0.59	0.59
	F (CV%)	14.8	14.8	14.8	14.8	14.8	14.8	14.8	14.8	14.8	5.50	5.50	5.50	5.50	5.50	5.50	5.50	5.50	5.50	14.8	14.8	14.8	14.8	14.8	14.8	14.8	14.8	14.8	5.50	5.50	5.50	5.50	5.50	5.50	5.50	5.50	5.50
After connection	T (s)	0.60	0.60	0.60	0.60	0.60	0.60	0.60	0.60	0.60	0.60	0.60	0.60	0.60	0.60	0.60	0.60	0.60	0.60	0.62	0.62	0.62	0.62	0.62	0.62	0.62	0.62	0.62	0.62	0.62	0.62	0.62	0.62	0.62	0.62	0.62	0.62
	F (CV%)	5.63	5.88	6.07	5.94	5.63	6.08	5.87	6.30	5.86	5.62	5.66	5.67	5.68	5.66	5.67	5.67	5.65	5.69	5.54	5.00	5.00	5.00	5.85	5.00	5.31	5.00	5.00	5.03	5.03	5.14	5.00	4.99	5.02	5.02	5.02	5.08
		Line like pattern																		Lattice like pattern 2																	
		cell-community									fast stable									cell-community									fast stable								
Before connection	T (s)	1.21	1.21	1.21	1.21	1.21	1.21	1.21	1.21	1.21	0.59	0.59	0.59	0.59	0.59	0.59	0.59	0.59	0.59	1.31	1.31	1.31	1.31	1.31	1.31	1.31	1.31	1.31	0.59	0.59	0.59	0.59	0.59	0.59	0.59	0.59	0.59
	F (CV%)	15.5	15.5	15.5	15.5	15.5	15.5	15.5	15.5	15.5	5.50	5.50	5.50	5.50	5.50	5.50	5.50	5.50	5.50	14.8	14.8	14.8	14.8	14.8	14.8	14.8	14.8	14.8	5.50	5.50	5.50	5.50	5.50	5.50	5.50	5.50	5.50
After connection	T (s)	0.61	0.61	0.61	0.61	0.60	0.60	0.60	0.60	0.60	0.60	0.60	0.60	0.60	0.60	0.60	0.60	0.60	0.60	0.63	0.63	0.63	0.63	0.63	0.63	0.63	0.63	0.63	0.63	0.63	0.63	0.63	0.63	0.63	0.63	0.63	0.63
	F (CV%)	14.4	11.5	8.86	9.31	6.53	5.79	5.91	5.64	6.13	5.34	5.35	5.36	5.38	5.38	5.36	5.37	5.41	5.48	5.05	5.05	5.05	5.05	5.05	5.05	5.05	5.05	5.05	5.05	5.05	5.19	5.04	5.04	5.10	5.05	5.08	5.18
		Radial like pattern																		Lattice like pattern 1																	
		cell-community									fast unstable									cell-community									fast unstable								
Before connection	T (s)	1.24	1.24	1.24	1.24	1.24	1.24	1.24	1.24	1.24	0.95	0.95	0.95	0.95	0.95	0.95	0.95	0.95	0.95	1.31	1.31	1.31	1.31	1.31	1.31	1.31	1.31	1.31	0.95	0.95	0.95	0.95	0.95	0.95	0.95	0.95	0.95
	F (CV%)	14.8	14.8	14.8	14.8	14.8	14.8	14.8	14.8	14.8	28.2	28.2	28.2	28.2	28.2	28.2	28.2	28.2	28.2	14.8	14.8	14.8	14.8	14.8	14.8	14.8	14.8	14.8	25.5	25.5	25.5	25.5	25.5	25.5	25.5	25.5	25.5
After connection	T (s)	1.09	1.09	1.09	1.09	1.09	1.09	1.09	1.09	1.09	1.09	1.09	1.09	1.09	1.09	1.09	1.09	1.09	1.09	1.13	1.13	1.13	1.13	1.13	1.13	1.13	1.13	1.13	1.13	1.13	1.13	1.13	1.13	1.13	1.13	1.13	1.13
	F (CV%)	14.2	14.2	14.2	14.2	14.2	14.2	14.2	14.2	14.2	14.2	14.2	14.2	14.2	14.2	14.2	14.2	14.2	14.2	14.0	14.0	14.0	14.0	14.0	14.0	14.0	14.0	14.0	14.0	14.0	14.0	14.0	14.0	14.0	14.0	14.0	14.0
		Line like pattern																		Lattice like pattern 2																	
		cell-community									fast unstable									cell-community									fast unstable								
Before connection	T (s)	1.21	1.21	1.21	1.21	1.21	1.21	1.21	1.21	1.21	0.84	0.84	0.84	0.84	0.84	0.84	0.84	0.84	0.84	1.31	1.31	1.31	1.31	1.31	1.31	1.31	1.31	1.31	0.95	0.95	0.95	0.95	0.95	0.95	0.95	0.95	0.95
	F (CV%)	15.5	15.5	15.5	15.5	15.5	15.5	15.5	15.5	15.5	27.2	27.2	27.2	27.2	27.2	27.2	27.2	27.2	27.2	14.8	14.8	14.8	14.8	14.8	14.8	14.8	14.8	14.8	25.5	25.5	25.5	25.5	25.5	25.5	25.5	25.5	25.5
After connection	T (s)	0.98	0.98	0.98	0.98	0.98	0.98	0.98	0.98	0.98	0.98	0.98	0.98	0.98	0.98	0.98	0.98	0.98	0.98	1.17	1.17	1.17	1.17	1.17	1.17	1.17	1.17	1.17	1.17	1.17	1.17	1.17	1.17	1.17	1.17	1.17	1.17
	F (CV%)	19.3	18.6	18.6	18.6	18.6	18.6	18.6	18.6	18.6	18.6	18.6	18.6	18.6	18.6	18.6	18.6	18.6	18.6	14.2	14.2	14.2	14.2	14.2	14.2	14.2	14.2	14.2	14.2	14.2	14.2	14.2	14.2	14.2	14.2	14.2	14.2
		Radial like pattern																		Lattice like pattern 1																	
		cell-community									slow stable									cell-community									slow stable								
Before connection	T (s)	1.24	1.24	1.24	1.24	1.24	1.24	1.24	1.24	1.24	1.84	1.84	1.84	1.84	1.84	1.84	1.84	1.84	1.84	1.31	1.31	1.31	1.31	1.31	1.31	1.31	1.31	1.31	1.91	1.91	1.91	1.91	1.91	1.91	1.91	1.91	1.91
	F (CV%)	14.8	14.8	14.8	14.8	14.8	14.8	14.8	14.8	14.8	9.45	9.45	9.45	9.45	9.45	9.45	9.45	9.45	9.45	14.8	14.8	14.8	14.8	14.8	14.8	14.8	14.8	14.8	9.24	9.24	9.24	9.24	9.24	9.24	9.24	9.24	9.24
After connection	T (s)	1.44	1.44	1.44	1.44	1.44	1.44	1.44	1.44	1.44	1.44	1.44	1.44	1.44	1.44	1.44	1.44	1.44	1.44	1.48	1.48	1.48	1.48	1.48	1.48	1.48	1.48	1.48	1.48	1.48	1.48	1.48	1.48	1.48	1.48	1.48	1.48
	F (CV%)	11.3	11.3	11.4	11.3	11.3	11.3	11.3	11.3	11.3	11.3	11.3	11.3	11.3	11.3	11.3	11.3	11.3	11.3	11.6	11.5	11.5	11.6	11.5	11.6	11.5	11.5	11.5	11.5	11.5	11.5	11.5	11.5	11.5	11.5	11.5	11.5
		Line like pattern																		Lattice like pattern 2																	
		cell-community									slow stable									cell-community									slow stable								
Before connection	T (s)	1.21	1.21	1.21	1.21	1.21	1.21	1.21	1.21	1.21	1.80	1.80	1.80	1.80	1.80	1.80	1.80	1.80	1.80	1.31	1.31	1.31	1.31	1.31	1.31	1.31	1.31	1.31	1.91	1.91	1.91	1.91	1.91	1.91	1.91	1.91	1.91
	F (CV%)	15.5	15.5	15.5	15.5	15.5	15.5	15.5	15.5	15.5	10.4	10.4	10.4	10.4	10.4	10.4	10.4	10.4	10.4	14.8	14.8	14.8	14.8	14.8	14.8	14.8	14.8	14.8	9.24	9.24	9.24	9.24	9.24	9.24	9.24	9.24	9.24
After connection	T (s)	1.31	1.31	1.31	1.31	1.31	1.31	1.31	1.31	1.31	1.31	1.31	1.31	1.31	1.31	1.31	1.31	1.31	1.31	1.60	1.60	1.60	1.60	1.60	1.60	1.60	1.60	1.60	1.60	1.60	1.60	1.60	1.60	1.60	1.60	1.60	1.60
	F (CV%)	15.2	15.2	15.2	15.2	15.2	15.2	15.2	15.2	15.2	15.2	15.2	15.2	15.2	15.2	15.2	15.2	15.2	15.2	9.51	9.48	9.47	9.50	9.47	9.52	9.47	9.47	9.47	9.48	9.47	9.48	9.47	9.48	9.48	9.47	9.47	9.47
		Radial like pattern																		Lattice like pattern 1																	
		cell-community									slow unstable									cell-community									slow unstable								
Before connection	T (s)	1.24	1.24	1.24	1.24	1.24	1.24	1.24	1.24	1.24	1.82	1.82	1.82	1.82	1.82	1.82	1.82	1.82	1.82	1.31	1.31	1.31	1.31	1.31	1.31	1.31	1.31	1.31	2.12	2.12	2.12	2.12	2.12	2.12	2.12	2.12	2.12
	F (CV%)	14.8	14.8	14.8	14.8	14.8	14.8	14.8	14.8	14.8	53.0	53.0	53.0	53.0	53.0	53.0	53.0	53.0	53.0	14.8	14.8	14.8	14.8	14.8	14.8	14.8	14.8	14.8	46.2	46.2	46.2	46.2	46.2	46.2	46.2	46.2	46.2
After connection	T (s)	1.19	1.19	1.19	1.19	1.19	1.19	1.19	1.19	1.19	1.19	1.19	1.19	1.19	1.19	1.19	1.19	1.19	1.19	1.28	1.28	1.28	1.28	1.28	1.28	1.28	1.28	1.28	1.28	1.28	1.28	1.28	1.28	1.28	1.28	1.28	1.28
	F (CV%)	14.9	14.9	14.9	14.9	14.9	14.9	14.9	14.9	14.9	14.9	14.9	14.9	14.9	14.9	14.9	14.9	14.9	14.9	14.8	14.8	14.8	14.8	14.8	14.8	14.8	14.8	14.8	14.8	14.8	14.8	14.8	14.8	14.8	14.8	14.8	14.8
		Line like pattern																		Lattice like pattern 2																	
		cell-community									slow unstable									cell-community									slow unstable								
Before connection	T (s)	1.21	1.21	1.21	1.21	1.21	1.21	1.21	1.21	1.21	1.69	1.69	1.69	1.69	1.69	1.69	1.69	1.69	1.69	1.31	1.31	1.31	1.31	1.31	1.31	1.31	1.31	1.31	2.12	2.12	2.12	2.12	2.12	2.12	2.12	2.12	2.12
	F (CV%)	15.5	15.5	15.5	15.5	15.5	15.5	15.5	15.5	15.5	51.9	51.9	51.9	51.9	51.9	51.9	51.9	51.9	51.9	14.8	14.8	14.8	14.8	14.8	14.8	14.8	14.8	14.8	46.2	46.2	46.2	46.2	46.2	46.2	46.2	46.2	46.2
After connection	T (s)	1.16	1.16	1.16	1.16	1.16	1.16	1.16	1.16	1.16	1.16	1.16	1.16	1.16	1.16	1.16	1.16	1.16	1.16	1.28	1.28	1.28	1.28	1.28	1.28	1.28	1.28	1.28	1.28	1.28	1.28	1.28	1.28	1.28	1.28	1.28	1.28
	F (CV%)	18.5	18.5	18.5	18.5	18.5	18.5	18.5	18.5	18.5	18.5	18.5	18.5	18.5	18.5	19.1	19.1	18.5	18.5	14.4	14.4	14.4	14.4	14.4	14.4	14.4	14.4	14.4	14.4	14.4	14.4	14.4	14.4	14.4	14.4	14.4	14.4

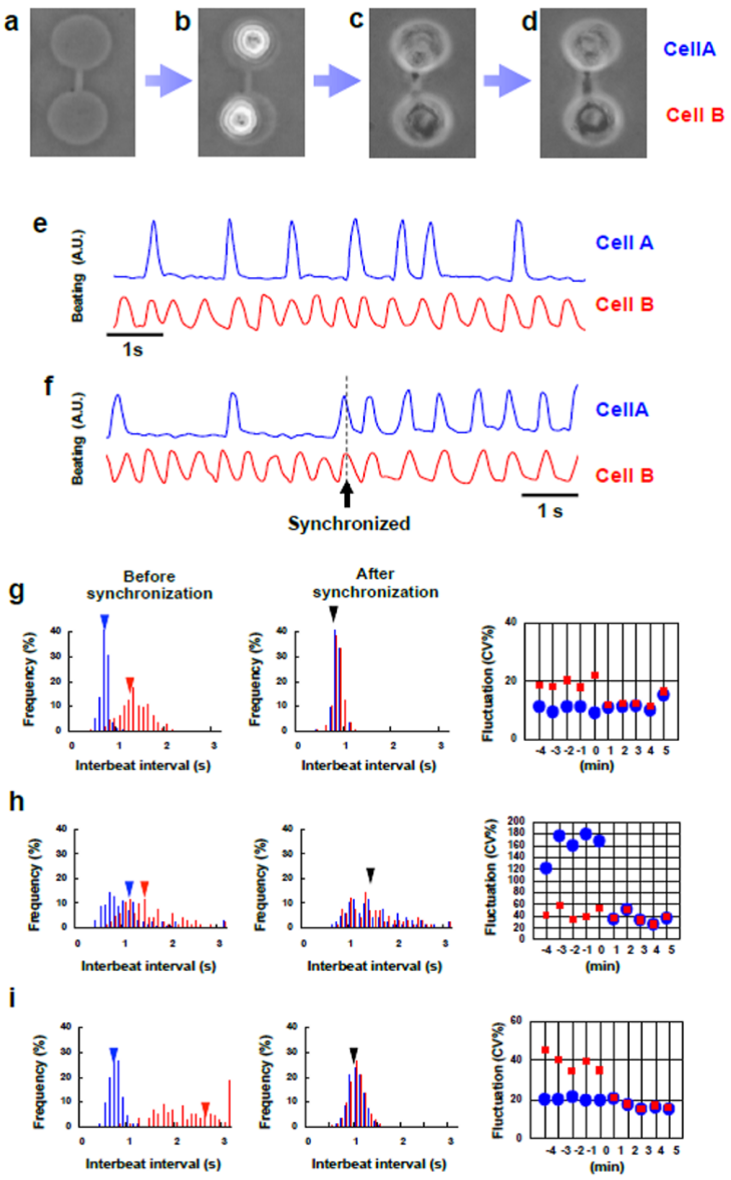

Supplementary Figure S1: Beating synchronization of a cardiomyocyte pair in a two well agarose microchamber obtained from a cluster. (a)-(d) Micrographs of two cardiomyocyte network formation; (a) agarose microchamber before cell cultivation, (b) two cardiomyocytes placed on each well, (c) a day after cultivation started (before network formation), (d) two cardiomyocytes connected through narrow micorochannel. (e)-(f) Time course of the beatings of cell A and cell B; before synchronization (e), and synchronization started(f). (g)-(i) Three types of distributions of interbeat intervals of two cardiomyocyte networks and the change of beating rhythm fluctuation before and after synchronization; (g) faster beating cell followed to slower beating cell, (h) slower beating cell followed to faster beating cell, (i) synchronized to new beating rhythm between two beating rythms. The data for the two cardiomyocytes are shown by blue and red bars, and the blue and red triangles show the corresponding mean values before synchronization, and the black triangles show those after synchronization. CV, coefficient of variation (100 × standard deviation/mean beat rate) (Kojima et al. [29]).

Supplementary Note S1: Determination of a free parameter μ. Let us define two indices, ΔT and ΔF, to evaluate the deviation of the theoretical values from the experimental values.

$$\Delta T := \sqrt{\frac{1}{26}\sum_{k=1}^{13}\left\{(T_k^1 - T_k^{\mathrm{ex}})^2 + (T_k^2 - T_k^{\mathrm{ex}})^2\right\}}, \tag{1}$$

$$\Delta F := \sqrt{\frac{1}{26}\sum_{k=1}^{13}\left\{(F_k^1 - F_k^{\mathrm{ex}})^2 + (F_k^2 - F_k^{\mathrm{ex}})^2\right\}}, \tag{2}$$

where T_k^{ex} denotes the experimental value of the average beating rhythm of the cell-i in the pair k after synchronization, and $T_k^i(i = 1, 2)$ denotes the corresponding theoretical value obtained by our model. Similarly F_k^{ex} denotes the experimental value of fluctuation (CV) of the cell-i in the pair k after synchronization, and $F_k^i(i = 1, 2)$ denotes its theoretical value. Supplementary Figures S2a and S2b show the dependence of ΔT and ΔF on μ. For $6 \lesssim \mu \lesssim 10$, ΔT keeps to take lowest value, while ΔF takes lowest value for $6 \lesssim \mu \lesssim 20$. Both of them are almost constants for fairly wide range of μ. This fact means that our model is robust against a free parameter μ.

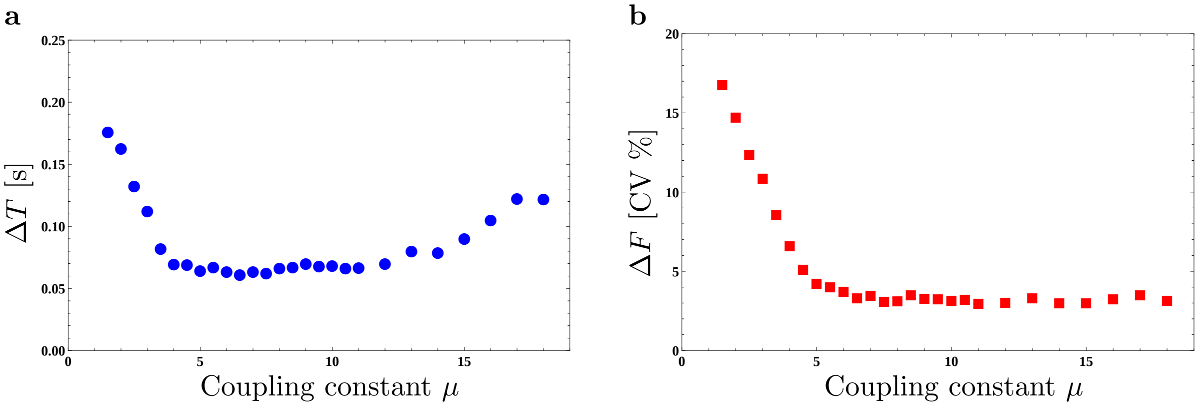

Supplementary Figure S2: Parameter μ dependence of deviation of numerical values from experimental data. The deviation between numerical simulation and experimental data is measured with the two quantities ΔT and ΔF by changing parameter μ.

Supplementary Note S2: Comparison of our model and Kuramoto model. When cell-1 which had a mean beating rhythm of 1.40s and fluctuation of 12.3 [CV%] and cell-2 which had a mean beating rhythm of 1.10s and fluctuation of 25.1 [CV%] were coupled, we found that the slow and stable cell (cell-1) acted as a pacemaker and the beating rhythm after synchronisation was tuned to this cardiomyocyte in our model (Supplementary Fig. S3). However, the Kuramoto model showed that beating fluctuation of the slow and stable cardiomyocyte (cell-1) was increased after synchronisation. The mean beating rate and beating fluctuation of our model and those of the Kuramoto model are shown in the Supplementary Table S9.

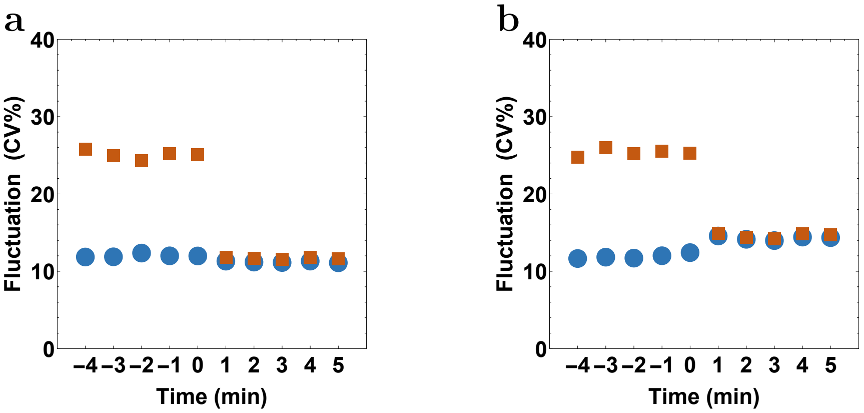

Supplementary Figure S3: Comparison of our model and Kuramoto model. The change in beating fluctuation before and after synchronisation is shown. The blue circles and brown squares represent the corresponding mean values for 1 min of beating fluctuation of cell-1 and cell-2, respectively. (a) The numerical result of our model, and (b) the numerical result of the Kuramoto model.

Supplementary Table S9: Comparison of our model and Kuramoto model. The symbol T_i and F_i denote the mean beating rate and the beating fluctuation of the cell-$i (i = 1, 2)$, respectively. The symbol T denotes the mean beating rate and F the beating fluctuation after synchronization.

	Before synchronization				After synchronization	
	T_1(s)	F_1(CV%)	T_2(s)	F_2(CV%)	T(s)	F(CV%)
Our model	1.40	12.3	1.10	25.1	1.30	11.6
Kuramoto model	1.40	12.3	1.10	25.1	1.23	14.6

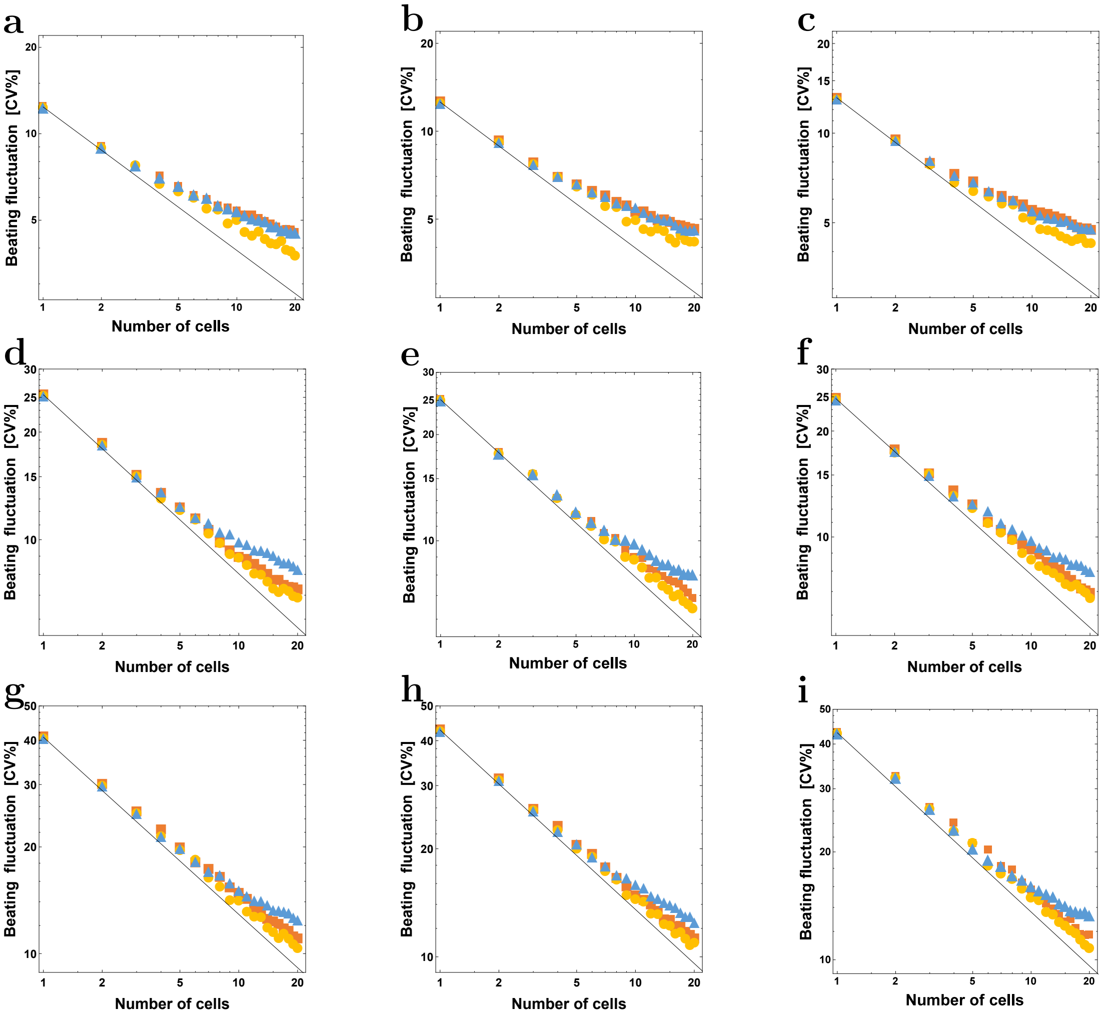

Supplementary Figure S4: Size dependence of fluctuation for three types of configuration. Size dependence of fluctuation is shown in double logarithmic graphs. The components of the network are model cardiomyocytes with the same characteristics. Brown squares indicate beating fluctuation [CV%] of cardiomyocytes in star network, orange circles indicate beating fluctuation in the 2D lattice network, and blue triangles indicate beating fluctuation in the 1D lattice network. Panels **a**–**c** show the size dependence of fluctuation for three types of configurations. CV values are set to 12.3 [CV%], and mean beating rhythms are different among (**a**)–(**c**): (**a**) a mean beating rhythm of 0.64s, (**b**) a mean beating rhythm of 1.25s, (**c**) a mean beating rhythm of 2.09s. Panels **d**–**f** show the size dependence of fluctuation for three types of configurations. CV values are set to 25.1 [CV%], and mean beating rhythms are different among (**d**)–(**f**): (**d**) a mean beating rhythm of 0.68s, (**e**) a mean beating rhythm of 1.23s, (**f**) a mean beating rhythm of 2.03s. Panels **g**–**i** show the size dependence of fluctuation for three types of configurations. CV values are set to 43.0 [CV%], and mean beating rhythms are different among (**g**)–(**i**): (**g**) a mean beating rhythm of 0.83 s, (**h**) a mean beating rhythm of 1.92 s, (**i**) a mean beating rhythm of 2.71s. The black straight line denotes $\propto N^{-1/2}$ where N is the number of cardiomyocytes in the network.

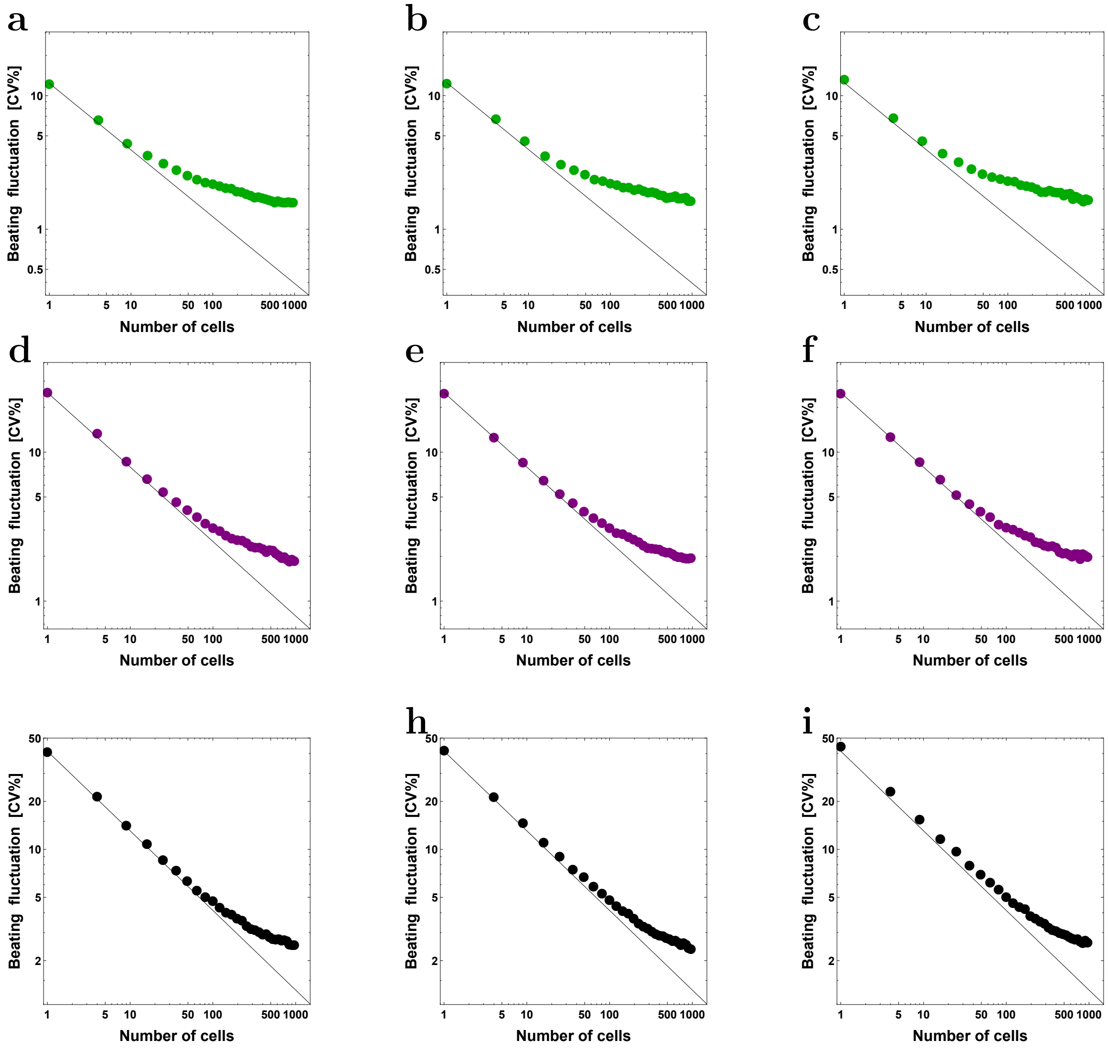

Supplementary Figure S5: Size dependence of fluctuation for a large 2D lattice network. Size dependence of fluctuation is shown in double logarithmic graphs. The components of the network are model cardiomyocytes with the same characteristics. Panels **a–c** show the size dependence of fluctuation for 2D lattice networks. CV values are set to 12.3 [CV%], and mean beating rhythms are different among (**a**)–(**c**): (**a**) a mean beating rhythm of 0.64s, (**b**) a mean beating rhythm of 1.25s, (**c**) a mean beating rhythm of 2.09s. Panels **d–f** show the size dependence of fluctuation for 2D lattice networks. CV values are set to 25.1 [CV%], and mean beating rhythms are different among (**d**)–(**f**): (**d**) a mean beating rhythm of 0.68s, (**e**) a mean beating rhythm of 1.23s, (**f**) a mean beating rhythm of 2.03s. Panels **g–i** show the size dependence of fluctuation for 2D lattice networks. CV values are set to 43.0 [CV%], and mean beating rhythms are different among (**g**)–(**i**): (**g**) a mean beating rhythm of 0.83 s, (**h**) a mean beating rhythm of 1.92 s, (**i**) a mean beating rhythm of 2.71s. The black straight line denotes $\propto N^{-1/2}$ where N is the number of cardiomyocytes in the network.
